# PAC1 constrains type 2 inflammation through promotion of CGRP signaling in ILC2s

**DOI:** 10.1172/JCI180109

**Published:** 2024-09-17

**Authors:** Yuan Jin, Bowen Liu, Qiuyu Li, Xiangyan Meng, Xiaowei Tang, Yan Jin, Yuxin Yin

**Affiliations:** 1 Institute of Systems Biomedicine, Department of Pathology, Beijing Key Laboratory of Tumor Systems Biology, School of Basic Medical Sciences, Peking University Health Science Center, Beijing, China.; 2 School of Medicine, Chinese University of Hong Kong (Shenzhen), Guangdong, China.; 3 Department of Respiratory and Critical Care Medicine, Peking University Third Hospital, Beijing, China.; 4 Peking-Tsinghua Joint Center for Life Sciences, Beijing, China.; 5 Institute of Precision Medicine, Peking University Shenzhen Hospital, Shenzhen, China.

**Keywords:** Immunology, Allergy, Innate immunity

## Abstract

Dysfunction of group 2 innate lymphoid cells (ILC2s) plays an important role in the development of type 2 inflammation–related diseases such as asthma and pulmonary fibrosis. Notably, neural signals are increasingly recognized as pivotal regulators of ILC2s. However, how ILC2s intrinsically modulate their responsiveness to these neural signals is still largely unknown. Here, using single-cell RNA-Seq, we found that the immune-regulatory molecule phosphatase of activated cells 1 (PAC1) selectively promoted the signaling of the neuropeptide calcitonin gene–related peptide (CGRP) in ILC2s in a cell-intrinsic manner. Genetic ablation of PAC1 in ILC2s substantially impaired the inhibitory effect of CGRP on proliferation and IL-13 secretion. PAC1 deficiency significantly exacerbated allergic airway inflammation induced by *Alternaria alternata* or papain in mice. Moreover, in human circulating ILC2s, the expression level of *PAC1* was also significantly negatively correlated with the number of ILC2s and their expression level of *IL13*. Mechanistically, PAC1 was necessary for ensuring the expression of CGRP response genes by influencing chromatin accessibility. In summary, our study demonstrated that PAC1 is an important regulator of ILC2 responses, and we propose that PAC1 is a potential target for therapeutic interventions in type 2 inflammation–related diseases.

## Abstract 2

PAC1 constrains ILC2 responses intrinsically by promoting CGRP signaling.

## Graphical abstract

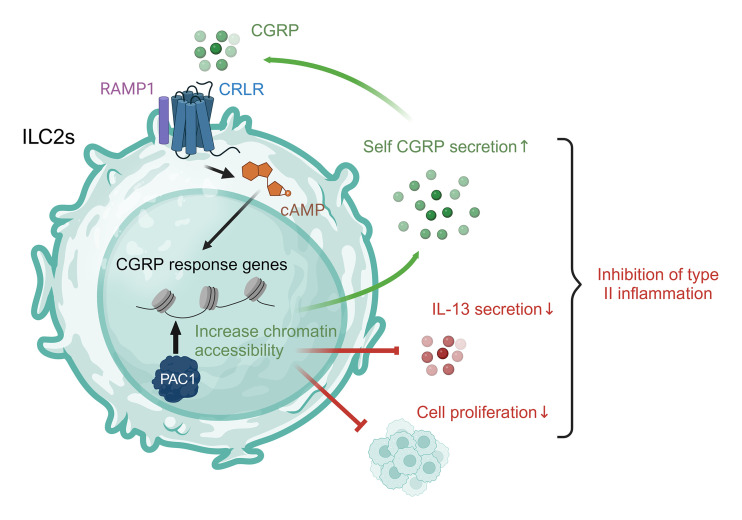

## Introduction

Type 2 inflammation is characterized by the secretion of type 2 cytokines (e.g., IL-4, IL-5, and IL-13) and the recruitment of eosinophils (1) and plays a core role in host antiparasitic immunity (2). However, excessive type 2 inflammation can lead to a range of pathological processes, with the most pronounced effect, including asthma (3–5) and pulmonary fibrosis (6–8), on the respiratory system. Among multiple cell types that are involved in inducing type 2 inflammation, RAG-deficient group 2 innate lymphoid cells (ILC2s) are increasingly being recognized as the most central contributors. When exposed to epithelium-derived, alarmin-like factors such as IL-33, IL-25, and thymic stromal lymphopoietin (TSLP), ILC2s can rapidly proliferate and produce large amounts of type 2 cytokines (mainly IL-5 and IL-13) prior to antigen presentation (9–12). Therefore, identifying molecules essential for the regulation of ILC2 responses is of great clinical importance for more accurate diagnoses and effective treatment of diseases related to type 2 inflammation.

Although they share the key transcription factor GATA3 with Th2 cells (13), as a group of tissue-resident innate immune cells, ILC2s possess a unique gene expression profile and are coregulated by complex signals in the mucosal microenvironment. Notably, accumulating evidence suggests that neurotransmitters and neuropeptides secreted by neurons or neuroendocrine cells play a major role in regulating ILC2 responses (14). Among them, the representative positive regulatory molecules include neuromedin U (NMU) (15–17), vasoactive intestinal peptide (VIP) (18), and acetylcholine (19, 20), whereas the representative negative regulatory molecules include calcitonin gene–related peptide (CGRP) (21–23), dopamine (24), and epinephrine (25). However, despite the gradually comprehensive description of a neuroregulatory network of ILC2s, the mechanisms by which ILC2s intrinsically maintain and coordinate their responses to these neural signals are still largely unknown.

Phosphatase of activated cells 1 (PAC1), also known as dual-specificity phosphatase 2 (DUSP2), belongs to the nuclear-localized DUSPs and is selectively expressed in immune cells, especially lymphoid cells (26–29). Our previous research has demonstrated the key role of PAC1 in limiting the effector functions of T cell subsets. In CD4^+^ T cells, PAC1 selectively constrains the differentiation of Th17 cells by dephosphorylating STAT3 (27), thus constraining autoimmune colitis. In CD8^+^ T cells, PAC1 is involved in reshaping chromatin accessibility in a phosphatase domain–independent manner, ultimately promoting the exhaustion of CD8^+^ T cells and attenuating host antitumor immunity (29). These findings suggest that PAC1 is a powerful intrinsic regulatory molecule of lymphoid cells and, moreover, functions through distinct mechanisms in different cell types. Nevertheless, the role of PAC1 in ILC subtypes and its involvement in the regulation of type 2 inflammation remain unexplored.

In this study, we demonstrated that PAC1 plays an essential role in controlling ILC2 responses and in a cell-intrinsic manner, thus inhibiting type 2 inflammation. Furthermore, utilizing single-cell RNA-Seq (scRNA-Seq), we showed that PAC1 selectively promoted CGRP signaling in ILC2s. Deletion of PAC1 impaired the inhibitory effect of CGRP on proliferation and IL-13 secretion of ILC2s both in vitro and in vivo. Mechanistically, we found that PAC1 was necessary for increasing the chromatin accessibility of CGRP-response genes in ILC2s. These observations establish a link between PAC1 and type 2 immunity, providing a potential target for therapeutic interventions of type 2 inflammation–related diseases. More than that, our research identifies PAC1 as a powerful intrinsic tool for ILC2s to modulate their responsiveness to neural signals.

## Results

### PAC1 constrains type 2 inflammation.

**Figure 1:**
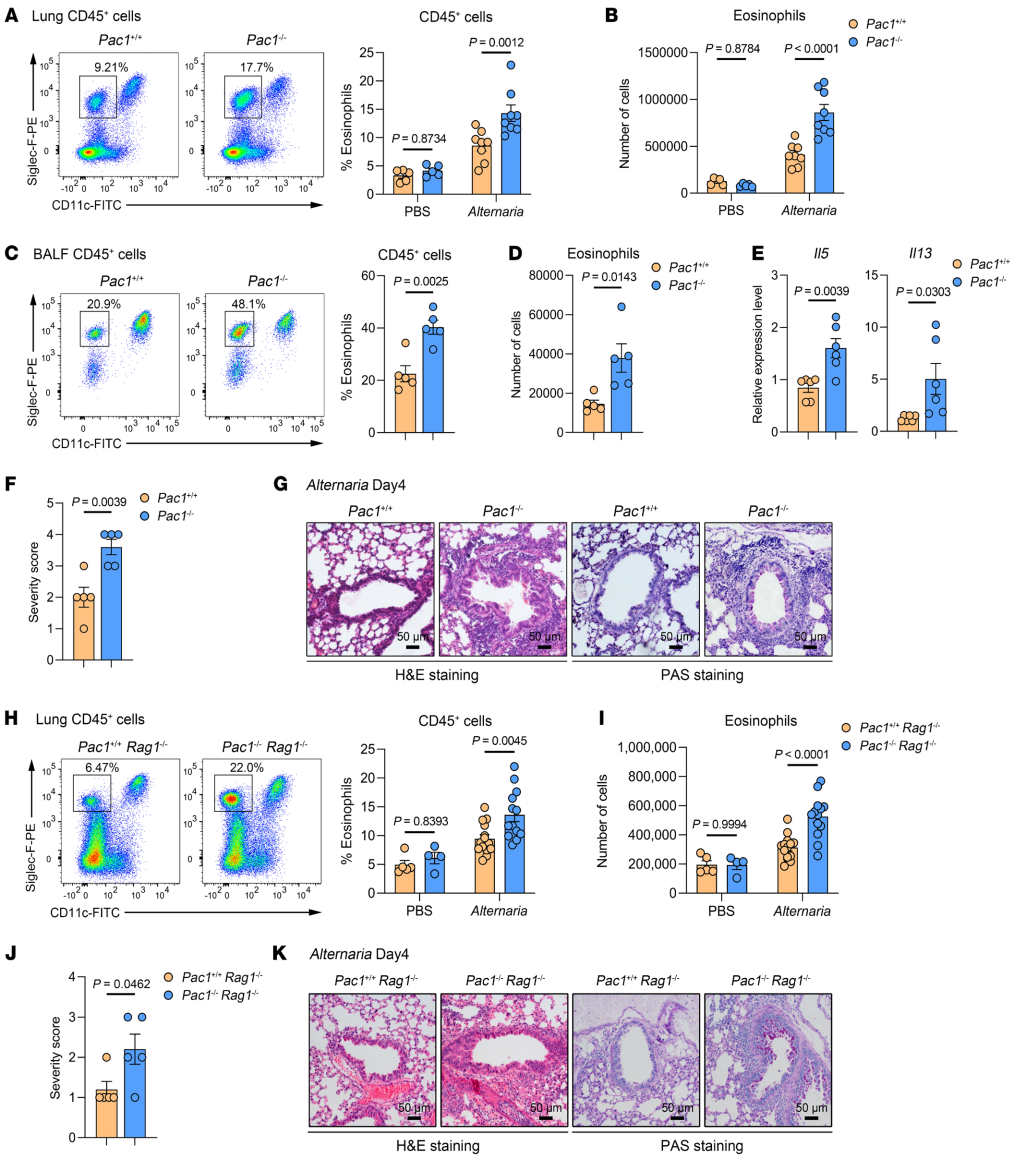
PAC1 constrains *A. alternata*–induced allergic airway inflammation in mice. (**A** and **B**) Frequency (**A**) and absolute number (**B**) of lung eosinophils in *Pac1^+/+^* and *Pac1^–/–^* mice on day 4 after PBS (*Pac1^+/+^*, *n* = 5; *Pac1^–/–^*, *n* = 5) or *A. alternata* (*Pac1^+/+^*, *n* = 8; *Pac1^–/–^*, *n* = 8) administration. (**C** and **D**) Frequency (**C**) and absolute number (**D**) of BALF eosinophils in *Pac1^+/+^* (*n* = 5) and *Pac1^–/–^* mice (*n* = 5) on day 4 after *A. alternata* administration. (**E**) *Il5* and *Il13* expression levels in lung tissues of *Pac1^+/+^* (*n* = 6) and *Pac1^–/–^* mice (*n* = 6) on day 4 after *A. alternata* administration. (**F**) Histological score for lungs from *Pac1^+/+^* (*n* = 5) and *Pac1^–/–^* mice (*n* = 5) on day 4 after *A. alternata* administration. (**G**) Representative H&E- and PAS-stained lung sections from *Pac1^+/+^* and *Pac1^–/–^* mice on day 4 after *A. alternata* administration. (**H** and **I**) Frequency (**H**) and absolute number (**I**) of lung eosinophils in *Pac1^+/+^ Rag1^–/–^* and *Pac1^–/–^* *Rag1^–/–^* mice on day 4 after PBS (*Pac1^+/+^ Rag1^–/–^*, *n* = 5; *Pac1^–/–^* *Rag1^–/–^*, *n* = 4) or *A. alternata* (*Pac1^+/+^ Rag1^–/–^*, *n* = 14; *Pac1^–/–^* *Rag1^–/–^*, *n* = 13) administration. (**J**) Histological score for lungs from *Pac1^+/+^ Rag1^–/–^* (*n* = 5) and *Pac1^–/–^* *Rag1^–/–^* mice (*n* = 5) on day 4 after *A. alternata* administration. (**K**) Representative H&E- and PAS-stained lung sections from *Pac1^+/+^ Rag1^–/–^* and *Pac1^–/–^* *Rag1^–/–^* mice on day 4 after *A. alternata* administration. Data are shown as the mean ± SEM. Statistical significance was assessed using 2-way ANOVA followed by Holm-Šidák multiple-comparison test (**A**, **B**, **H**, and **I**) or 2-tailed, unpaired Student’s *t* test (**C**–**F** and **J**). Scale bars: 50 μm (**G** and **K**).

To elucidate the effect of PAC1 in type 2 inflammation, the fungal extract *Alternaria alternata* was used to challenge WT and *Pac1-*KO mice to induce allergic airway inflammation. Following nasal administration of *A. alternata* once a day for 4 consecutive days, we observed that *Pac1^–/–^* mice had a significant increase in the proportion and absolute number of eosinophils in lung tissue in comparison with WT mice (Figure 1, A and B, and Supplemental Figure 1A; supplemental material available online with this article; https://doi.org/10.1172/JCI180109DS1). Additionally, *Pac1^–/–^* mice displayed a greater infiltration of eosinophils into bronchoalveolar lavage fluid (BALF) when compared with their WT counterparts (Figure 1, C and D). Higher levels of type 2 cytokine (*Il5*, *Il13*) mRNAs in the lung tissues of *Pac1^–/–^* mice were also observed (Figure 1E). Furthermore, periodic acid–Schiff (PAS) and H&E staining revealed an overall more severe airway inflammation in *Pac1^–/–^* mice following *A. alternata* challenge (Figure 1F) that was specifically characterized by greater proliferation of goblet cells and increased infiltration of inflammatory cells (Figure 1G). Consistent with these findings, in a separate allergic airway inflammation model induced by nasal challenge with papain, *Pac1^–/–^* mice also had more severe airway inflammation relative to WT mice, as demonstrated by flow cytometric analyses and H&E staining (Supplemental Figures 1, B–E). Collectively, these results provide initial evidence suggesting that PAC1 plays an important role in suppressing type 2 inflammation.

Type 2 inflammation involves both innate and adaptive immune cells, with Th2 cells being the primary mediators of type 2 immune responses among adaptive immune cells. Intriguingly, a prior study from our laboratory reported that in CD4^+^ T cells, PAC1 selectively restricts the differentiation of Th17 cells by dephosphorylating STAT3, rather than by participating in the regulation of Th2 cells polarization (27). To investigate further whether the regulation of type 2 inflammation by PAC1 is independent of adaptive immunity, we crossbred *Pac1^+/+^* and *Pac1^–/–^* mice with *Rag1^–/–^* mice to generate *Pac1^+/+^ Rag1^–/–^* and *Pac1^–/–^* *Rag1^–/–^* mice. After subsequent challenge with *A. alternata*, *Pac1^–/–^* *Rag1^–/–^* mice still exhibited significantly greater infiltration of eosinophils in lung than did *Pac1^+/+^ Rag1^–/–^* mice (Figure 1, H and I). Furthermore, *Pac1^–/–^* *Rag1^–/–^* mice also displayed increased histological injury in lung (Figure 1, J and K). These results provide additional evidence that PAC1 indeed exerted a constraining effect on type 2 inflammation through innate immunity.

### PAC1 exhibits suppressive effects on ILC2 responses.

**Figure 2:**
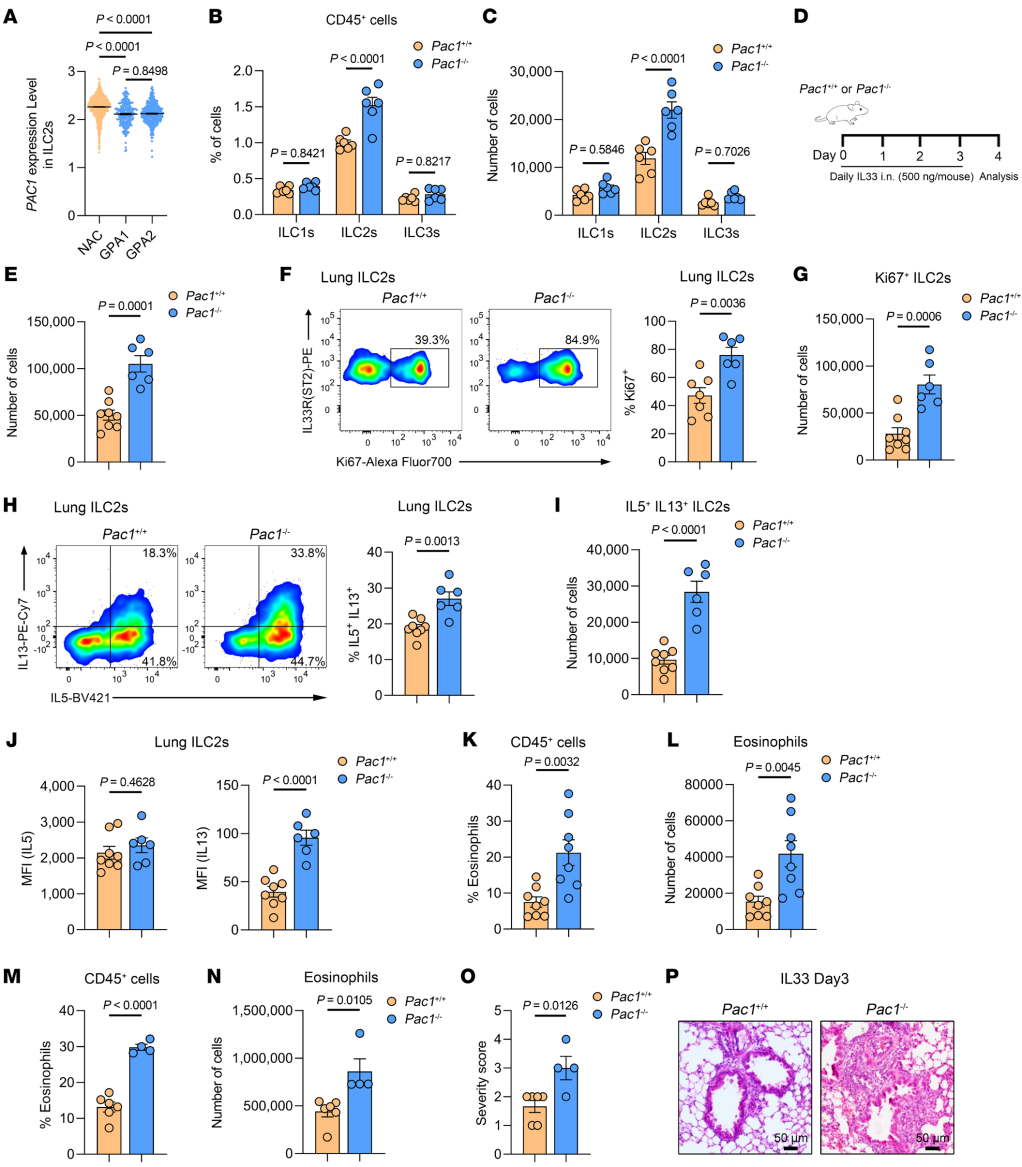
PAC1 exhibits a suppressive effect on ILC2 responses. (**A**) Violin plot of *PAC1* expression levels in human ILC2s sorted from peripheral blood of patients with GPAs and NACs. The scCITE-Seq data were obtained from GSE163367 (30). (**B** and **C**) Frequency (**B**) and absolute number (**C**) of lung T-bet^+^ ILC1s, GATA3^+^ ILC2s, and RORγT^+^ ILC3s from *Pac1^+/+^* (*n* = 6) and *Pac1^–/–^* mice (*n* = 6) in the cell resting states. (**D**) Experimental protocol followed for in vivo ILC2 activation using IL-33. The data shown in **E**–**P** were obtained on day 4 after IL-33 administration. (**E**) Absolute number of lung ILC2s in *Pac1^+/+^* (*n* = 8) and *Pac1^–/–^* mice (*n* = 6). (**F** and **G**) Frequency (**F**) and absolute number (**G**) of lung Ki67^+^ ILC2s from *Pac1^+/+^* (*n* = 7) and *Pac1^–/–^* mice (*n* = 6). (**H** and **I**) Frequency (**H**) and absolute number (**I**) of lung IL-5^+^IL-13^+^ ILC2s from *Pac1^+/+^* (*n* = 8) and *Pac1^–/–^* mice (*n* = 6). (**J**) MFI of IL-5 or IL-13 in lung ILC2s from *Pac1^+/+^* (*n* = 8) and *Pac1^–/–^* mice (*n* = 6). (**K** and **L**) Frequency (**K**) and absolute number (**L**) of BALF eosinophils from *Pac1^+/+^* (*n* = 8) and *Pac1^–/–^* mice (*n* = 8). (**M** and **N**) Frequency (**M**) and absolute number (**N**) of lung eosinophils from *Pac1^+/+^* (*n* = 6) and *Pac1^–/–^* mice (*n* = 4). (**O**) Histological score for lungs from *Pac1^+/+^* (*n* = 6) and *Pac1^–/–^* mice (*n* = 4). (**P**) Representative images of H&E-stained lung sections from *Pac1^+/+^* and *Pac1^–/–^* mice. Scale bars: 50 μm. Data are shown as the mean ± SEM. Statistical significance was assessed using 1-way ANOVA followed by Tukey’s multiple-comparison test (**A**), 2-way ANOVA followed by Holm-Šidák multiple-comparison test (**B** and **C**), or 2-tailed, unpaired Student’s *t* test (**E**–**O**).

Among innate immune cells, ILC2s are increasingly recognized as core contributors in the induction of type 2 inflammation. We first assessed the expression level of *Pac1* in various types of cells isolated from the lungs of normal WT mice. The results indicated that *Pac1* tends to be expressed in lymphoid immune cells (including ILC2s) in comparison with myeloid immune cells and nonimmune cells, although the *Pac1* expression levels in innate lymphoid cells were indeed lower than in adaptive lymphoid cells (Supplemental Figure 2A). To explore whether PAC1 has a potential role in regulating ILC2 functions, we firstly used scCITE-Seq data (sourced from GSE163367) provided by Golebski et al. (30) and compared *PAC1* expression levels in human peripheral blood–derived ILC subsets in individuals with grass-pollen allergies (GPAs) and nonallergic healthy controls (NACs). The results showed that *PAC1* expression was significantly reduced in ILC2s from individuals with GPAs relative to those from NACs (Figure 2A). According to the research of Golebski et al., ILC2s in GPAs exhibit a significant increase in both the number and proportion of IL-13^+^ clusters. This finding suggested that PAC1 might serve as a constraining factor limiting the capacity of ILC2s to drive type 2 inflammation in clinical allergic disease, initially supporting the idea that PAC1 may be involved in regulating ILC2 responses.

To systematically investigate the effect of PAC1 on ILC2s, we initially compared the proportions and absolute numbers of ILC subsets in the lungs of WT and *Pac1*-KO mice in their resting states (gating strategy shown in Supplemental Figure 2B). The results indicated that, even in the absence of stimulation, PAC1 deficiency significantly increased both the proportion (gated on CD45^+^ immune cells) and absolute number of GATA3^+^ ILC2s in lung, whereas those of T-bet^+^ ILC1s and RORγT^+^ ILC3s were not affected (Figure 2, B and C). Similarly, in the resting state, mice lacking PAC1 also exhibited a significant increase in GATA3^+^ ILC2s in other tissues where ILC2s are enriched, including epididymal adipose tissue, intestinal lamina propria, and colonic lamina propria (Supplemental Figure 2C), suggesting that the requirement of PAC1 for maintenance of ILC2 homeostasis does not involve tissue heterogeneity. We subsequently analyzed the protein levels of a series of ILC2-related surface markers, including GATA3, IL-33R (ST2), SCA-1, KLRG1, IL-7Rα (CD127), Thy-1, and IL-25R (IL-17RB), in lung ILC2s from WT and *Pac1^–/–^* mice. The results showed that, in the resting state, the vast majority of lung ILC2s in both WT and *Pac1^–/–^* mice were ST2^+^KLRG1^+^IL-25R^lo^ “natural” ILC2s, and the expression level of each mentioned ILC2-related marker was not affected by PAC1 deficiency (Supplemental Figures 2, D and E), suggesting that the ablation of PAC1 did not alter the phenotype of ILC2s in tissues. On the basis of these findings, we first suspected that PAC1 deficiency may affect the developmental processes of ILC2s. Interestingly, the proportions of common lymphoid progenitors (CLPs) (defined as lineage^–^SCA-1^–^CD127^hi^c-Kit^lo^), common ILCs progenitors (CHILPs) (defined as lineage^–^CD127^hi^α4β7^+^FLT3^–^CD25^–^), and ILC2ps (defined as lineage^–^CD127^+^CD25^+^ST2^+^GATA3^+^) in murine bone marrow were all comparable in WT and *Pac1^–/–^* mice (Supplemental Figure 2F). In addition, the absence of PAC1 did not affect the levels of GATA3 protein in bone marrow ILC2ps either (Supplemental Figure 2G).

Next, we investigated the effect of PAC1 on ILC2 responses under inflammatory conditions. WT and *Pac1^–/–^* mice were intranasally challenged with IL-33, a key epithelium-derived alarmin known to induce the expansion and functional activation of ILC2s, once a day for 4 consecutive days (Figure 2D). As anticipated, we observed that the absolute number of lung ILC2s in *Pac1^–/–^* mice consistently exceeded that of WT mice upon IL-33 treatment (Figure 2E). Besides, during IL-33 stimulation, lung ILC2s in both WT and *Pac1^–/–^* mice still were observed to be ST2^+^ “natural” ILC2s, with comparable expression levels of various ILC2-related surface markers (Supplemental Figure 3, A and B). Notably, we also detected a greater number of Ki67^+^ ILC2s in the lungs of *Pac1^–/–^* mice (Figure 2, F and G, and Supplemental Figure 3C), indicative of enhanced ILC2 proliferation. Additionally, upon IL-33 challenge, both the proportion and absolute number of IL-5^+^ and IL-13^+^ ILC2s in the lungs of *Pac1^–/–^* mice were significantly higher than those in WT mice (Figure 2, H and I, and Supplemental Figure 3C). It is interesting to note that in the flow cytometry experiments, *Pac1^–/–^* ILC2s had higher levels (MFI) of IL-13 but not IL-5 (Figure 2J), suggesting that PAC1 mainly constrained IL-13 secretion. Furthermore, most likely as a consequence of these augmented ILC2 responses, the proportion and the absolute number of eosinophils in BALF (Figure 2, K and L) and lungs (Figure 2, M and N) were all higher in *Pac1^–/–^* mice. Exacerbated airway inflammation in *Pac1^–/–^* mice was also detected by histological staining (Figure 2, O and P).

Furthermore, studies have shown that IL-25 can induce the generation of a population of inflammatory ILC2s (iILC2s) characterized by ST2^–^KLRG1^hi^IL-25R^+^ in lungs (18). Thus, WT and *Pac1^–/–^* mice were also challenged with IL-25 once a day for 4 consecutive days. The results indicated that both WT and *Pac1^–/–^* mice were able to generate iILC2s in lungs, with comparable expression levels of KLRG1 and IL-25R detected in WT and *Pac1^–/–^* iILC2s (Supplemental Figure 3, D and E). However, both the proportion and absolute number of iILC2s in the lungs of *Pac1^–/–^* mice were significantly higher than those in WT mice (Supplemental Figure 3, F and G).

Taken together, our data indicate that the negative regulation of ILC2 responses by PAC1 is broadly suitable and determined only by cell identity, lacking both tissue specificity and subset specificity. Additionally, at least according to the analyses of lung ILC2s, we found no evidence indicating that PAC1 was involved in affecting the phenotype or heterogeneity of ILC2s.

### PAC1 plays a cell-intrinsic inhibitory role in ILC2s.

**Figure 3:**
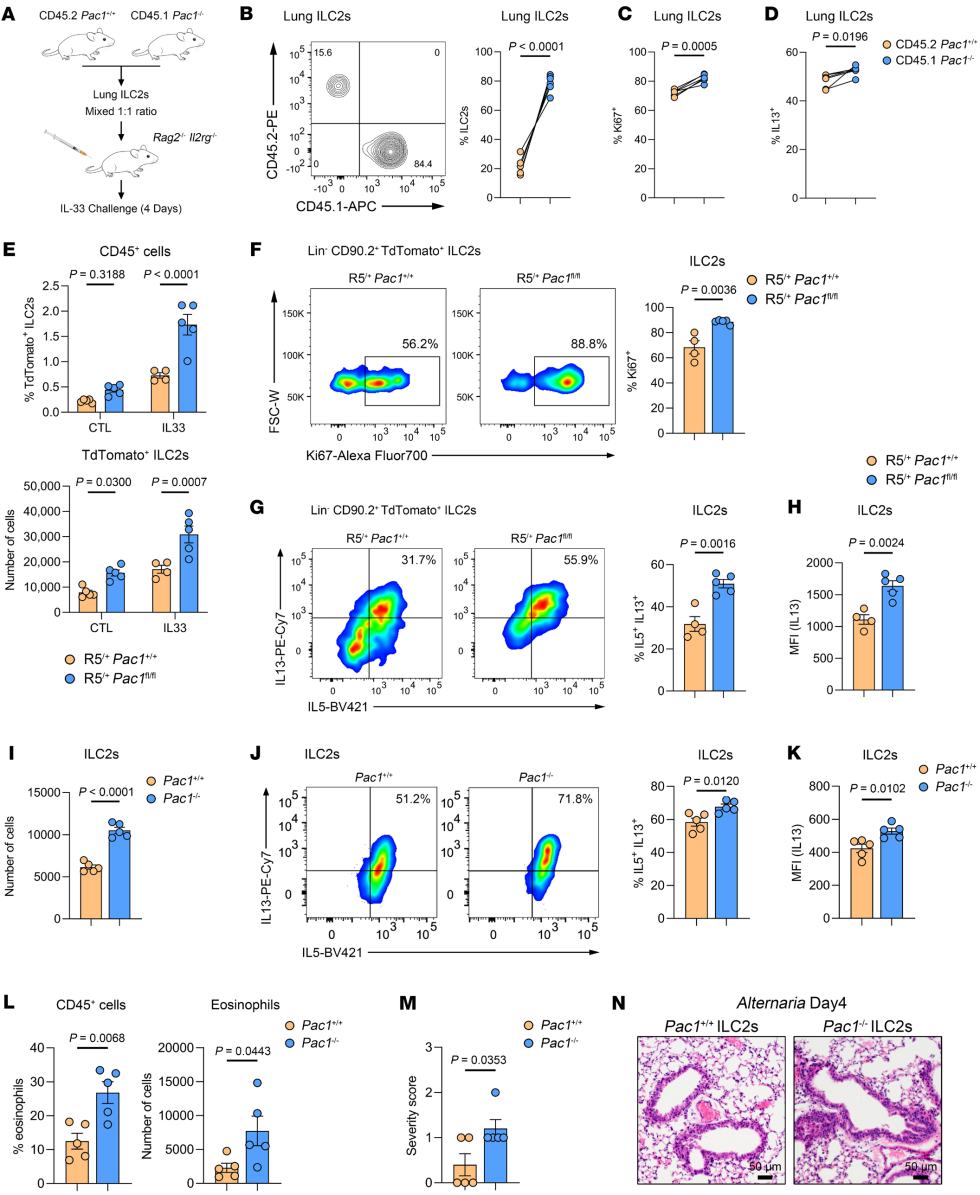
PAC1 plays a cell-intrinsic inhibitory role in ILC2s. (**A**) Experimental protocol for adoptive transfer of mixed ILC2s into *Rag2^–/–^* *Il2rg^–/–^* mice. The data in **B**–**D** were obtained in recipient mice (*n* = 6) on day 4 after IL-33 administration. (**B**) Frequency of lung CD45.2^+^ *Pac1^+/+^* ILC2s and CD45.1^+^ *Pac1^–/–^* ILC2s. (**C**) Frequency of lung CD45.2^+^ *Pac1^+/+^* Ki67^+^ ILC2s and CD45.1^+^ *Pac1^–/–^* Ki67^+^ ILC2s. (**D**) Frequency of lung CD45.2^+^ *Pac1^+/+^* IL-13^+^ ILC2s and CD45.1^+^ *Pac1^–/–^* IL-13^+^ ILC2s. (**E**) Frequency and absolute number of lung ILC2s from R5^/+^ *Pac1^+/+^* and R5^/+^ *Pac1^fl/fl^* mice in the cell resting state (R5^/+^ *Pac1^+/+^*, *n* = 5; R5^/+^ *Pac1^fl/fl^*, *n* = 5) or on day 4 after IL-33 administration (R5^/+^ *Pac1^+/+^*, *n* = 4; R5^/+^ *Pac1^fl/fl^*, *n* = 5). (**F**–**H**) Frequency of lung Ki67^+^ ILC2s (**F**), frequency of lung IL-5^+^ IL-13^+^ ILC2s (**G**), and MFI of IL-13 in lung ILC2s (**H**) from R5^/+^ *Pac1^+/+^* (*n* = 4) and R5^/+^ *Pac1^fl/fl^* mice (*n* = 5) on day 4 after IL-33 administration. The data shown in **I**–**N** were obtained on day 4 after *A. alternata* administration. (**I**–**M**) Absolute number of lung ILC2s (**I**), frequency of lung IL-5^+^ IL-13^+^ ILC2s (**J**), MFI of IL-13 in lung ILC2s (**K**), frequency and absolute number of BALF eosinophils (**L**), and histological score for lungs (**M**) in recipient *Rag2^–/–^* *Il2rg^–/–^* mice, which had received equal numbers of *Pac1^+/+^* lung ILC2s (*n* = 5) or *Pac1^–/–^* lung ILC2s (*n* = 5). (**N**) Representative H&E-stained lung sections from recipient *Rag2^–/–^* *Il2rg^–/–^* mice, which had received equal numbers of *Pac1^+/+^* lung ILC2s or *Pac1^–/–^* lung ILC2s. Scale bars: 50 μm. Data are shown as the mean ± SEM. Statistical significance was assessed using a 2-tailed paired Student’s *t* test (**B**–**D**), 2-way ANOVA followed by Holm-Šidák multiple-comparison test (**E**), or 2-tailed, unpaired Student’s *t* test (**F**–**M**). Scale bars: 50 μm.

To further investigate whether the suppressive effect of PAC1 on ILC2 responses relies on the presence of other cell types, lung ILC2s sorted from CD45.2^+^ WT mice and CD45.1^+^ *Pac1^–/–^* mice (Supplemental Figure 4A) were mixed at a 1:1 ratio, followed by adoptive transfer into *Rag2^–/–^* *Il2rg^–/–^* mice, which are deficient in all lymphoid cells. The recipient mice were then intranasally challenged with IL-33 once a day for 4 consecutive days (Figure 3A). As expected, CD45.1^+^ *Pac1^–/–^* ILC2s accounted for a significantly higher proportion of all lung ILC2s compared with CD45.2^+^ WT ILC2s (Figure 3B). In addition, the percentages of Ki67^+^ cells (Figure 3C) and IL-13^+^ cells (Figure 3D) in CD45.1^+^ *Pac1^–/–^* ILC2s were significantly higher than those in CD45.2^+^ WT ILC2s. We then used a mixed bone marrow chimeric model to further investigate the cell-intrinsic inhibitory role of PAC1 in ILC2s. Bone marrow cells from CD45.2^+^ WT mice and CD45.1^+^ *Pac1^–/–^* mice were mixed at a 1:1 ratio (Supplemental Figure 4B), followed by adoptive transfer into *Rag2^–/–^* *Il2rg^–/–^* mice, which had been subjected to myeloablation by busulfan treatment. After an 8-week reconstitution period, the recipient mice were then subjected to an intranasal IL-33 challenge (Supplemental Figure 4C). As observed previously, in the lungs of the chimeric mice, CD45.1^+^ *Pac1^–/–^* ILC2s were present at a higher proportion (Supplemental Figure 4D) and displayed more robust proliferation (Supplemental Figure 4E) and greater IL-13 secretion than did CD45.2^+^ WT ILC2s (Supplemental Figure 4F). Furthermore, we generated *Pac1^fl/fl^* mice and crossbred them with B6(C)-*Il5*^tm1.1(icre)Lky/^J mice (18) (Red5 mice) to selectively delete *Pac1* in ILC2s (designated as R5^/+^ *Pac1^fl/fl^* mice). In line with the observations made in *Pac1^–/–^* mice, the absolute number of ILC2s in R5^/+^ *Pac1^fl/fl^* mice were higher than those in their R5^/+^ *Pac1^+/+^* littermates, even in the resting state (Figure 3E). Following IL-33 challenge, lung ILC2s in R5^/+^ *Pac1^fl/fl^* mice were still present in greater quantities (Figure 3E) and showed enhanced proliferation (Figure 3F and Supplemental Figure 4G) and increased IL-13 production (Figure 3, G and H, and Supplemental Figure 4G) compared with their R5^/+^ *Pac1^+/+^* littermates. In addition, we also confirmed exacerbated airway inflammation in R5^/+^ *Pac1^fl/fl^* mice by eosinophil assessments in BALF and lung tissues (Supplemental Figure 4, H and I). Finally, to further elucidate the pathological significance of the ablation of PAC1 in ILC2s, equal numbers of lung ILC2s from WT or *Pac1^–/–^* mice were adoptively transferred into recipient *Rag2^–/–^* *Il2rg^–/–^* mice, followed by the induction of allergic airway inflammation by nasal administration of *A. alternata*. As expected, the recipient mice that received *Pac1^–/–^* ILC2s exhibited a higher number (Figure 3I) and stronger IL-13 secretion ability (Figure 3, J and K) of lung ILC2s. Additionally, the proportion and absolute number of eosinophils infiltrating into BALF were both significantly higher in mice that received *Pac1^–/–^* ILC2s compared with those that received WT ILC2s (Figure 3L). Furthermore, mice that received *Pac1^–/–^* ILC2s also showed increased histological injury in lungs (Figure 3, M and N). This model indicated that the specific absence of PAC1 in ILC2s was sufficient to exacerbate type 2 inflammation. Taken together, these observations underscore a cell-intrinsic inhibitory role of PAC1 in modulating ILC2 responses.

### PAC1 deficiency impairs CGRP signaling in ILC2s.

**Figure 4:**
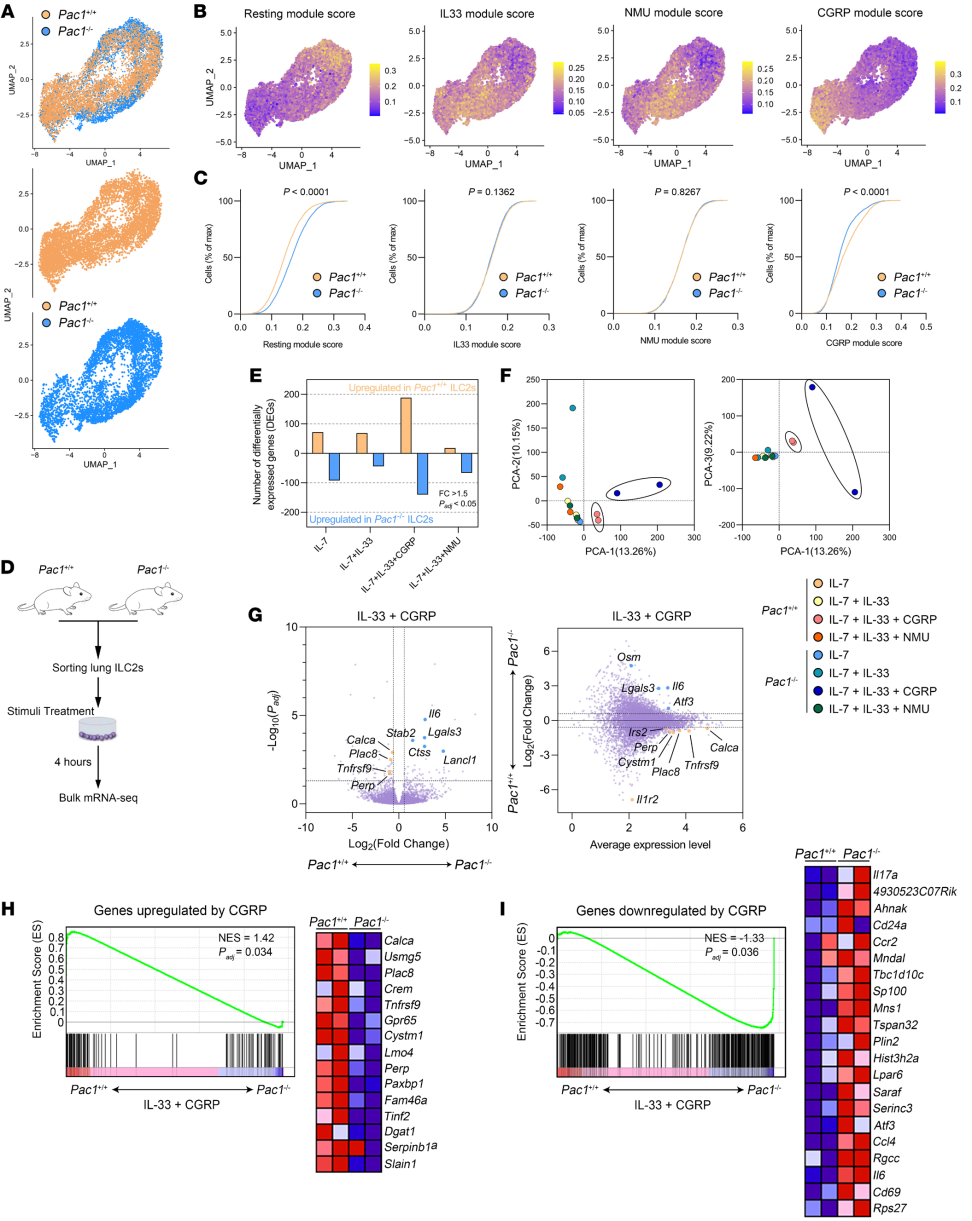
PAC1 deficiency impairs CGRP signaling in ILC2s. (**A**) UMAP plots of *Pac1^+/+^* and *Pac1^–/–^* lung ILC2s. (**B** and **C**) All *Pac1^+/+^* and *Pac1^–/–^* lung ILC2s were scored using gene sets induced, respectively, by IL-33, NMU, or CGRP. Genes that were downregulated by all 3 stimuli were also used to score the cells, marking ILC2s in the resting state. The score results are displayed on the UMAP plots (**B**), and the empirical cumulative distribution functions (ECDFs) separated by 2 genotypes are shown (**C**). max, maximum. (**D**) Experimental protocol followed for bulk mRNA-Seq analyses of *Pac1^+/+^* and *Pac1^–/–^* lung ILC2s after in vitro treatments. (**E**) Number of DEGs in *Pac1^+/+^* versus *Pac1^–/–^* lung ILC2s under different stimulation conditions (fold change >1.5; *P_adj_* < 0.05). (**F**) PCA of transcriptomes of *Pac1^+/+^* and *Pac1^–/–^* lung ILC2s under different stimulation conditions. Two replicates were analyzed per condition. (**G**) Volcano plot (left) and minus-versus-add (MA) plot (right) of DEGs in *Pac1^+/+^* versus *Pac1^–/–^* lung ILC2s after treatment with IL-33 plus CGRP (fold change >1.5; *P_adj_* < 0.05). M value = log_10_(average gene expression level); A value = log_2_(fold change [KO group/WT group]). Representative DEGs are shown. (**H** and **I**) GSEA was performed on *Pac1^+/+^* and *Pac1^–/–^* lung ILC2s after treatment with IL-33 plus CGRP, using gene sets upregulated (**H**) or downregulated (**I**) by CGRP (21). NES, normalized enrichment score.

To elucidate the molecular functions of PAC1 in ILC2s, we used the 10X Genomics platform to perform droplet-based scRNA-Seq on lung ILC2s purified from WT and *Pac1^–/–^* mice. After initial filtering, 5,429 and 4,280 suitable lung ILC2s from WT and *Pac1^–/–^* mice, respectively, were retained and aggregated for subsequent analyses. The expression levels of some classic marker genes of ILC2s (*Gata3*, *Il1rl1*) and other ILC subsets (*Eomes*, *Tbx21*, *Rorc*, *Ncr1*) were initially examined in these cells to ensure the accuracy of the cell-sorting process (Supplemental Figure 5A). In the uniform manifold approximation and projection (UMAP), ILC2s from WT and *Pac1^–/–^* mice displayed a transcriptional continuum and could be divided into distinct subgroups (Figure 4A). To understand and elucidate the differences in transcriptional dynamics between WT and *Pac1^–/–^* ILC2s, all cells were scored using gene sets induced, respectively, by IL-33, NMU, or CGRP (21). Among them, IL-33 and NMU are well-established, potent activators of ILC2s, while CGRP has been identified in recent years as having a key role in the negative regulation of ILC2 responses. Additionally, gene sets that are downregulated by all 3 stimuli were also used to score the cells, marking ILC2s in the resting state. Notably, the scoring results indicated that the CGRP signaling was significantly suppressed in *Pac1^–/–^* ILC2s, whereas no difference in the activity levels of the IL-33 and NMU signaling pathways between WT and *Pac1^–/–^* ILC2s was observed (Figure 4, B and C). Meanwhile, the proportion of cells in the resting state was significantly higher in *Pac1^–/–^* ILC2s than in WT ILC2s, reflecting to some extent a hyperproliferative state of *Pac1^–/–^* ILC2s (Figure 4, B and C). Moreover, by observing the distribution differences of the 4 gene sets across the “cell cloud,” we thought that the CGRP regulation primarily targets ILC2s that have been activated by IL-33 or NMU. In other words, the CGRP pathway appears to act as a surveillant of the ILC2 activation process. This hypothesis was further supported by pseudotime analysis using the Monocle 3 R package (Supplemental Figure 5B). Collectively, the scRNA-Seq data initially suggest that PAC1 may constrain ILC2 responses by promoting CGRP signaling.

To provide additional evidence supporting the assertion that PAC1 enhances CGRP signaling in ILC2s, lung ILC2s sorted from WT and *Pac1^–/–^* mice were treated with IL-7, IL-7 plus IL-33, IL-7 plus IL-33 plus CGRP, or IL-7 plus IL-33 plus NMU for 4 hours, and then bulk mRNA-Seq was performed (Figure 4D). The results obtained showed that in the CGRP-treated group, there was a higher number of differentially expressed genes (DEGs) (fold change >1.5; adjusted *P* value [*P_adj_*] < 0.05) in WT versus *Pac1^–/–^* ILC2s compared with other treatment groups (Figure 4E). Principal component analysis (PCA) further showed that the global transcriptomic profiles of WT and *Pac1^–/–^* ILC2s exhibited greater divergence following CGRP stimulation (Figure 4F), indicating a more pronounced effect of PAC1 deficiency on CGRP signaling. Specifically, upon CGRP treatment, the expression levels of several CGRP-induced genes, including *Calca*, *Gpr65*, *Plac8*, *Tnfrsf9*, and *Perp*, were higher in WT ILC2s. Conversely, genes that promote inflammation and cell proliferation, such as *Il6*, *Lgals3*, and *Atf3*, were more highly expressed in *Pac1^–/–^* ILC2s (Figure 4G). These representative DEGs suggest that PAC1 deficiency does indeed suppress normal transduction of the CGRP pathway. Gene set enrichment analysis (GSEA) provided further support for this conclusion by demonstrating a significant enrichment of genes upregulated or downregulated by CGRP in WT and *Pac1^–/–^* ILC2s, respectively (Figure 4, H and I). Moreover, we also confirmed the promotion of cAMP-dependent GPCR signaling and the suppression of cell proliferation in WT ILC2s by GSEA (Supplemental Figure 5D). Taken together, these results indicated that loss of PAC1 disrupted the normal responsiveness of ILC2s to CGRP. Furthermore, our mRNA-Seq results again reaffirmed that NMU is essential to the mass secretion of effector cytokines (including IL-13, IL-9, and IL-17A) from ILC2s (Supplemental Figure 5, E and F). Interestingly, in the NMU-treated group, loss of PAC1 was also associated with elevated *Il13* expression levels (Supplemental Figure 5, E and F). This observation was consistent with our aforementioned results that PAC1 can inhibit IL-13 secretion. Although we cannot exclude the possibility that PAC1 may be directly involved in suppressing the NMU effect, it seems that the continuous reduced secretion of CGRP (corresponding to lower expression of *Calca*) by *Pac1^–/–^* ILC2s themselves is also an important contributor to this outcome.

### PAC1 promotes CGRP-mediated suppression on ILC2 responses.

**Figure 5:**
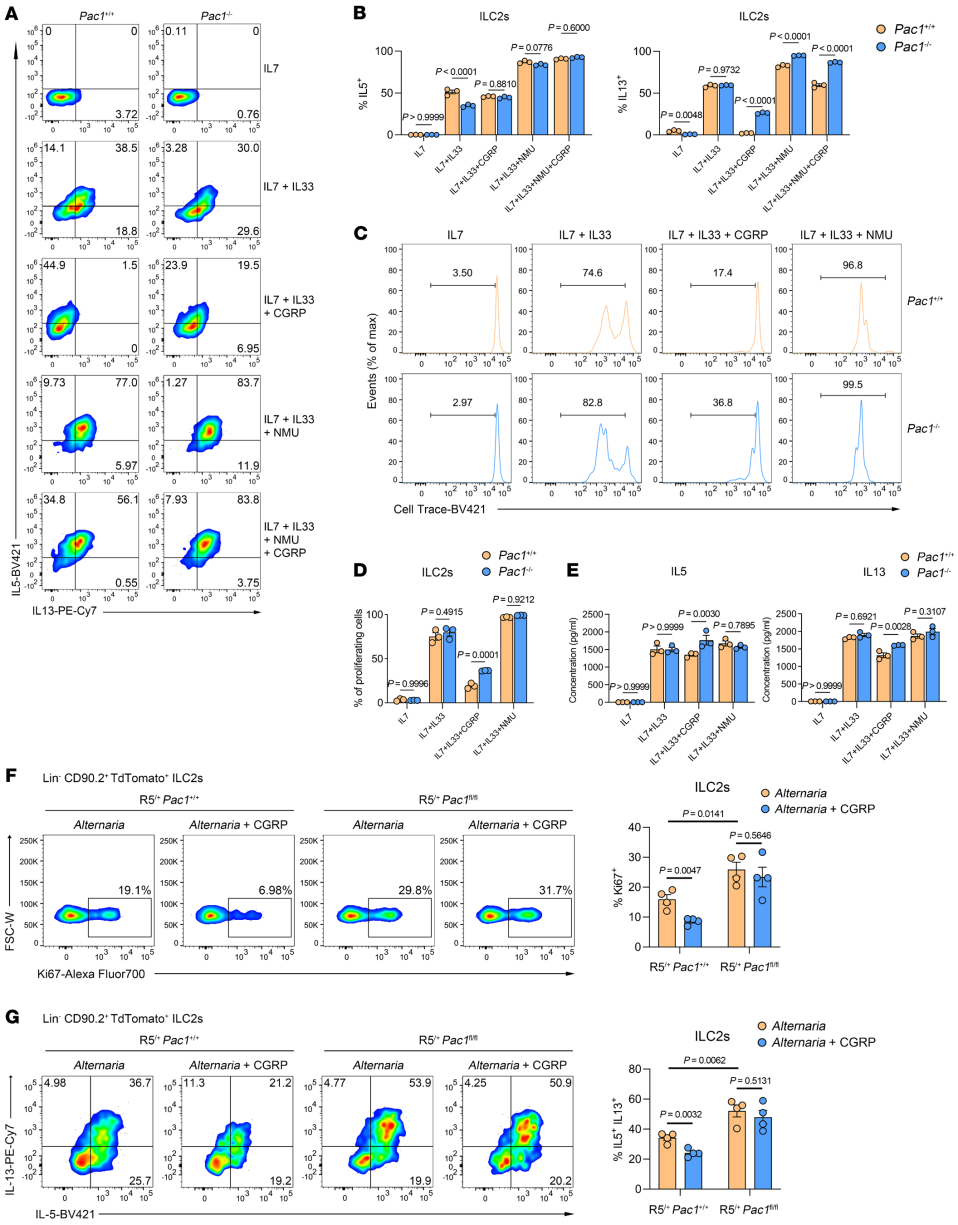
PAC1 promotes CGRP-mediated inhibition of ILC2 responses. (**A** and **B**) Purified *Pac1^+/+^* and *Pac1^–/–^* murine lung ILC2s were treated with IL-7 alone and in several combinations with IL-33, CGRP, and/or NMU for 4 hours, and the frequency of IL-5^+^ ILC2s and IL-13^+^ ILC2s was determined by flow cytometry. Three replicates were analyzed per condition. (**C** and **D**) Purified *Pac1^+/+^* and *Pac1^–/–^* murine lung ILC2s were labeled with CellTrace Violet and exposed to different conditions of stimulation for 3 days, and then the frequency of proliferating ILC2s was analyzed by flow cytometry. Three replicates were analyzed per condition. (**E**) Purified *Pac1^+/+^* and *Pac1^–/–^* murine lung ILC2s were treated under different conditions of stimulation for 3 days, and then the concentrations of a range of cytokines in the cell supernatants were assessed by Luminex liquid chip technology. Three replicates were analyzed per condition. (**F**) Frequency of lung Ki67^+^ ILC2s from R5^/+^ *Pac1^+/+^* and R5^/+^ *Pac1^fl/fl^* mice on day 4 after *A. alternata* administration (R5^/+^ *Pac1^+/+^*, *n* = 4; R5^/+^ *Pac1^fl/fl^*, *n* = 4) or *A. alternata* plus CGRP administration (R5^/+^ *Pac1^+/+^*, *n* = 4; R5^/+^ *Pac1^fl/fl^*, *n* = 4), as determined by flow cytometry. (**G**) Frequency of lung IL-5^+^ IL-13^+^ ILC2s from R5^/+^ *Pac1^+/+^* and R5^/+^ *Pac1^fl/fl^* mice on day 4 after *A. alternata* administration (R5^/+^ *Pac1^+/+^*, *n* = 4; R5^/+^ *Pac1^fl/fl^*, *n* = 4) or *A. alternata* plus CGRP administration (R5^/+^ *Pac1^+/+^*, *n* = 4; R5^/+^ *Pac1^fl/fl^*, *n* = 4), as determined by flow cytometry. Data are shown as the mean ± SEM. Statistical significance was assessed using 2-way ANOVA followed by Holm-Šidák multiple-comparison test (**B**–**E**) or 2-tailed, unpaired Student’s *t* test (**F** and **G**).

To provide experimental evidence that PAC1 promotes CGRP-mediated inhibition of ILC2 responses, purified lung ILC2s from WT and *Pac1^–/–^* mice were subjected to various combinations of IL-33, CGRP, or NMU for 4 hours in vitro, followed by the assessment of IL-5 and IL-13 protein levels by flow cytometry (Supplemental Figure 6A). The results unequivocally showed that CGRP-induced suppression of IL-13 secretion was significantly more pronounced in WT ILC2s than in with *Pac1^–/–^* ILC2s (Figure 5, A and B). In contrast, PAC1 deficiency appeared to have a minimal effect on IL-5 secretion following various stimulations (Figure 5, A and B). When the treatment period was extended to 3 days, the proliferation rates of WT and *Pac1^–/–^* ILC2s were compared. CGRP exerted a greater constraint on the expansion of WT ILC2s (Figure 5, C and D). In addition, when the Luminex liquid chip technology was used to simultaneously assess a range of cytokines in the supernatants after stimulation with CGRP, the concentrations of IL-5 and IL-13, as well as of a series of proinflammatory cytokines (including IL-9, IL-6, IL-1β, IL-18, IL-12) in WT ILC2s were all lower than those in *Pac1^–/–^* ILC2s (Figure 5E and Supplemental Figure 6B). Finally, we performed remedial nasal administration of CGRP to *A. alternata*–challenged R5^/+^ *Pac1^fl/fl^* mice and their R5^/+^ *Pac1^+/+^* littermates. The results showed that the addition of CGRP could indeed inhibit proliferation and IL-13 secretion of lung ILC2s and decrease infiltration of eosinophils into BALF in vivo, but only in R5^/+^ *Pac1^+/+^* mice (Figure 5, F and G, and Supplemental Figure 6, C and D).

Taken together, the above results substantiate the conclusion that PAC1 can promote CGRP signaling in ILC2s and thus plays a critical role in maintaining the inhibitory effect of CGRP on ILC2 responses.

### PAC1 promotes the expression of CGRP-response genes in ILC2s by influencing chromatin accessibility.

**Figure 6:**
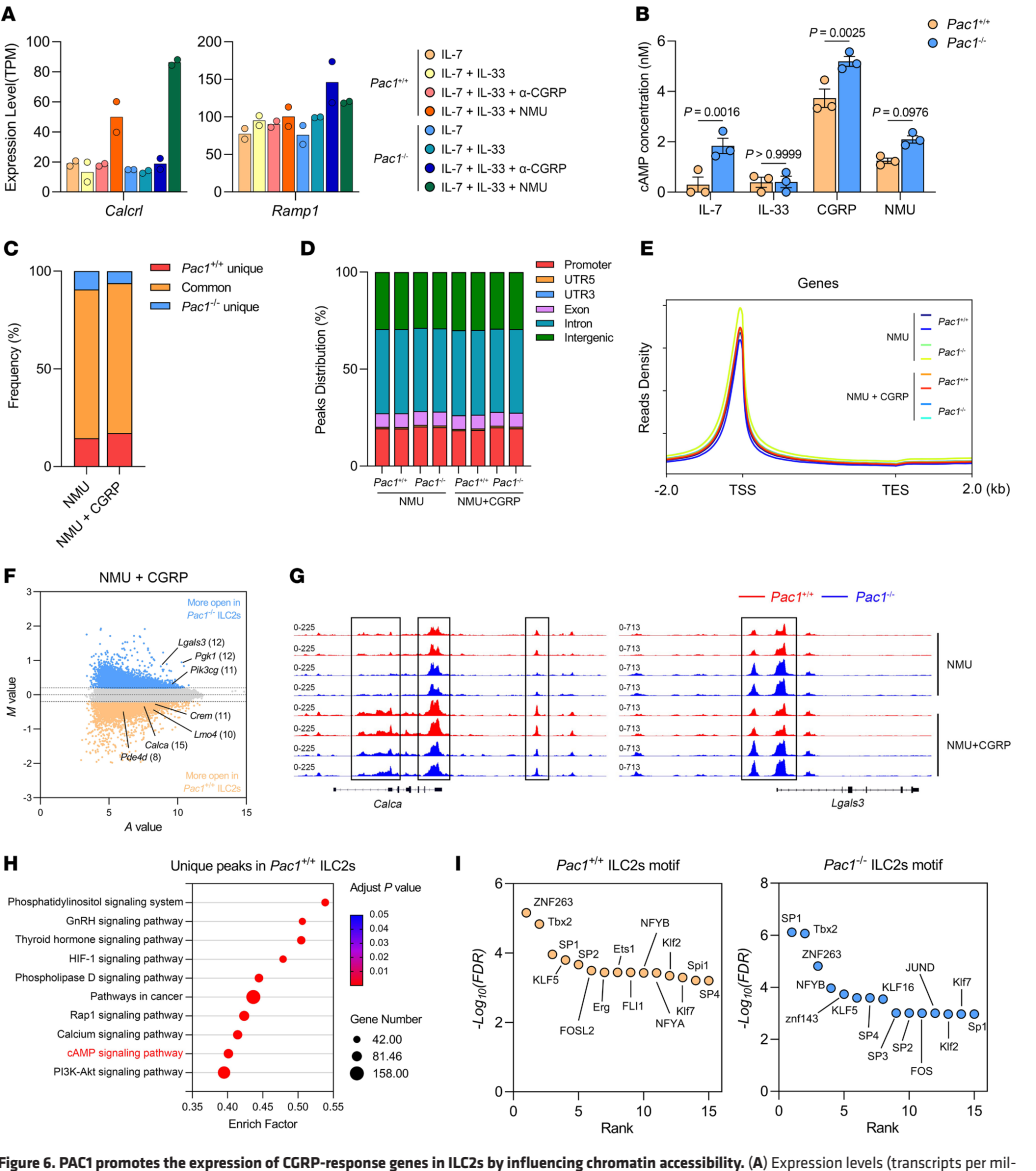
PAC1 promotes the expression of CGRP-response genes in ILC2s by influencing chromatin accessibility. (**A**) Expression levels (transcripts per million [TPM]) of *Calcrl* and *Ramp1* in *Pac1^+/+^* and *Pac1^–/–^* ILC2s under 4 different conditions of stimulation. Two replicates were analyzed per condition. (**B**) cAMP concentrations (nM) in cell lysates of *Pac1^+/+^* and *Pac1^–/–^* ILC2s after 20 minutes of treatment under the conditions shown, as determined by ELISA (mean ± SEM). Three replicates were analyzed per condition. Statistical significance was assessed using a 2-way ANOVA followed by Holm-Šidák multiple-comparison test. (**C**) Proportion of ATAC-Seq peaks shared by *Pac1^+/+^* and *Pac1^–/–^* ILC2s and the ATAC-Seq peaks unique to *Pac1^+/+^* or *Pac1^–/–^* ILC2s, respectively, after 4 hours of treatment with NMU or NMU plus CGRP. (**D**) Distribution of ATAC-Seq peaks across the genome in *Pac1^+/+^* and *Pac1^–/–^* ILC2s after 4 hours of treatment with NMU or NMU plus CGRP. (**E**) Distribution of ATAC-Seq signal around the TSS in *Pac1^+/+^* and *Pac1^–/–^* ILC2s after 4 hours of treatment with NMU or NMU plus CGRP. (**F**) MA plots of merged ATAC-Seq peaks (localized in promoter and exon regions) in *Pac1^+/+^* and *Pac1^–/–^* ILC2s after 4 hours of treatment with NMU plus CGRP. The top 5% of peaks (*P* < 0.05) for *Pac1^+/+^* or *Pac1^–/–^* ILC2s are highlighted. Representative genes are shown, with the number of peaks in parentheses. (**G**) Chromatin accessibility for the *Calca* and *Lgals3* loci in *Pac1^+/+^* and *Pac1^–/–^* ILC2s after 4 hours of treatment with NMU or NMU plus CGRP. (**H**) KEGG pathways enriched by genes containing ATAC-Seq peaks unique to *Pac1^+/+^* ILC2s after 4 hours of treatment with NMU plus CGRP. (**I**) Transcription factor binding motifs significantly enriched (top 15, *P* < 0.05) in the promoter regions and exon regions of *Pac1^+/+^* and *Pac1^–/–^* ILC2s.

To further investigate the mechanism by which PAC1 promotes CGRP signaling in ILC2s, we compared the expression levels of *Calcrl* and *Ramp1* (which encode CGRP receptor subunits) in WT and *Pac1^–/–^* ILC2s. The results showed that PAC1 deficiency did not affect the expression of *Calcrl* or *Ramp1*, and the extent of upregulation of *Calcrl* expression in *Pac1^–/–^* ILC2s was even greater than that in WT ILC2s when stimulated by NMU (Figure 6A). Additionally, in vitro assays demonstrated that PAC1 deficiency did not impede CGRP-induced cAMP production (Figure 6B), suggesting that PAC1 was not involved in maintaining the initial responsiveness of ILC2s to CGRP. Given our earlier findings that PAC1 alters chromatin accessibility during CD8^+^ T cell activation (29), fast ATAC-Seq of NMU-treated or NMU- plus CGRP-treated WT and *Pac1^–/–^* ILC2s were subsequently performed. Initial statistics showed that over 75% of the open regions were shared between WT and *Pac1^–/–^* ILC2s (Figure 6C), regardless of whether CGRP was included in the stimulus. Additionally, in each sample, the distribution of open chromatin regions across the genome and the overall enrichment signals at transcription start sites (TSSs) showed no significant differences (Figure 6, D and E). These analyses indicated that deletion of PAC1 did not alter the cell identity of ILC2s, which is consistent with what our previous results showed. Next, we only selected the peaks localized in promoter regions and exon regions for further differential analysis. Interestingly, following the addition of CGRP, many downstream genes induced by the CGRP/cAMP axis (including *Calca*, *Creb3l*, *Crem*, *Pde4d*, *Lmo4*, *Icos*) exhibited significantly (*P* < 0.05) more chromatin accessibility in WT ILC2s (Figure 6, F and G). On the other hand, deficiency of PAC1 also led to increased chromatin accessibility of some proinflammatory or proproliferative genes, including *Lgals3*, *Nlrp3*, and *Pik3cg* (Figure 6, F and G). In addition, Kyoto Encyclopedia of Genes and Genomes (KEGG) enrichment confirmed that, following CGRP stimulation, genes related to the unique peaks in WT ILC2s can be enriched in the cAMP signaling pathway (Figure 6H). To investigate the underlying reasons for these differences, we further assessed the enrichment levels of DNA binding motifs for each transcription factor in the promoter and exon regions of WT and *Pac1^–/–^* ILC2s. The Multiple Em for Motif Enrichment (MEME) motif analysis results showed that, during CGRP stimulation, the most enriched transcription factors (top 15) in WT ILC2s included several members of the ETS transcription factor family (such as ETS1, ERG1, FLI1), which was consistent with a previously reported finding that CGRP can selectively enhance the accessibility of ETS family transcription factors (21) (Figure 6I). However, this phenomenon was not observed in *Pac1^–/–^* ILC2s, and, meanwhile, members of the zinc finger transcription factor family (including SP1, SP3, KLF16) appeared to have stronger activity. Collectively, we propose that in ILC2s, PAC1 can indeed promote the expression of downstream genes induced by CGRP by influencing chromatin accessibility and potentially by enhancing the accessibility of ETS family members.

### Low expression of PAC1 in human ILC2s is associated with increased numbers and enhanced function of ILC2s.

**Figure 7:**
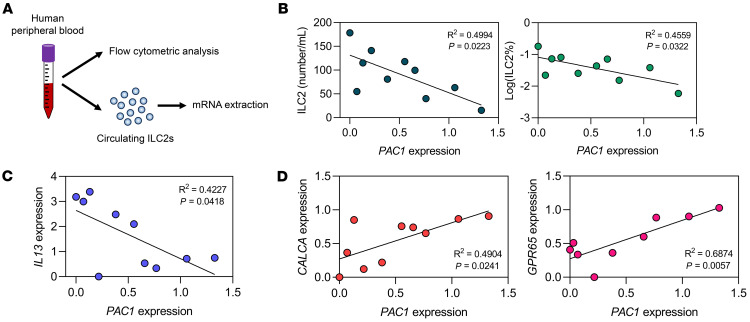
Low expression of *PAC1* in human ILC2s is associated with increased numbers and enhanced function of ILC2s. (**A**) Experimental protocol for the analyses of circulating ILC2s in human peripheral blood samples. (**B**) Correlation between *PAC1* expression levels in circulating ILC2s and the concentration (left) or the frequency (right) of circulating ILC2s. (**C**) Correlation between the *PAC1* expression levels and *IL13* expression levels in circulating ILC2s. (**D**) Correlation between the *PAC1* expression levels and *CALCA* expression levels or *GPR65* expression levels in circulating ILC2s.

Finally, to find clues indicating that the inhibitory effect of PAC1 identified in murine ILC2s is also applicable to human ILC2s, we randomly collected human peripheral blood samples and analyzed the cell concentration of circulating ILC2s (lineage^–^CD127^+^CRTH2^+^) in each donor (Figure 7A). Furthermore, circulating ILC2s from each donor were sorted for mRNA extraction, and expression levels of selected genes were assessed by qPCR (Figure 7A). Strikingly, the results showed that the expression level of *PAC1* in circulating ILC2s was significantly negatively correlated with either the cell concentration of circulating ILC2s (Figure 7B) or the expression level of *IL13* (Figure 7C). On the other hand, the *PAC1* expression level was significantly positively correlated with the expression levels of *CALCA* and *GPR65*, which are 2 of the most prominent genes induced by CGRP (Figure 7D). In summary, these correlation analyses suggest, to some extent, that PAC1 may also serve as a potential suppressor of human ILC2 responses and that it appears to play a consistent facilitating role in the CGRP signaling pathway in human ILC2s.

## Discussion

Inappropriate type 2 inflammation is strongly related to many pathological processes, including parasitosis, asthma, and pulmonary fibrosis. Research over the past decade has made it increasingly apparent that ILC2s are critically important players in the induction of type 2 inflammation. Therefore, identifying molecules essential for the regulation of development and function of ILC2s is of great significance for a full understanding and, ultimately, accurate diagnosis and effective treatment of type 2 inflammation–related diseases. In this study, we have detected substantially lower *PAC1* expression levels in ILC2s from patients with clinical allergies by analyzing scCITE-Seq data from the Gene Expression Omnibus (GEO) database. Subsequently, using experimental models of allergic airway inflammation, an ILC2 adoptive transfer model, and conditional *Pac1*-KO mice, we have rigorously demonstrated that PAC1 robustly inhibited ILC2 responses through a cell-intrinsic mechanism, thereby establishing it as a key molecule in the suppression of type 2 inflammation.

Among various external signals regulating ILC2 responses, increasing evidence suggests that neural signals play a decisive role. Among them, CGRP, by binding to receptors and promoting intracellular second-messenger cAMP production, exerts potent inhibition of proliferation and IL-13 secretion of ILC2s. However, beyond acting as a crucial negative regulator of ILC2s, CGRP is also involved in other physiological processes, including the mediation of sympathetic outflow, vasodilation, and wound healing (31). Therefore, the regulation of CGRP on ILC2s not only depends on changes in CGRP concentration in the microenvironment but also relies on careful modulation of the responsiveness of ILC2s to CGRP. Our study, utilizing scRNA-Seq and bulk mRNA-Seq, provides what we believe to be the first evidence that PAC1 is an important intrinsic molecule that selectively promotes CGRP signaling in ILC2s. PAC1 deficiency led to the failure of CGRP to inhibit ILC2 responses both in vitro and in vivo, resulting in excessive type 2 inflammation. Simply, PAC1 is a determining factor of ILC2 responsiveness to CGRP. Additionally, according to our sequencing analyses, the expression of gene *Calca*, which encodes CGRP, was among the genes most significantly influenced downstream by PAC1. This suggested that PAC1 was involved in ensuring the production of ILC2-derived CGRP during CGRP stimulation, thus sustaining this unique self-positive feedback loop. We propose that PAC1 in ILC2s might play a special role in the comprehensive biological regulatory network of CGRP. Moreover, it remains an important question for future exploration whether there are differences between immune cell–derived CGRP and neuron-derived CGRP, and whether PAC1, which is expressed exclusively in immune cells, is involved in this process. Finally, according to our study, we think that knowledge of the genetic status and expression levels of *PAC1* in patients with dysregulated ILC2 responses may also aid in assessing the possible effectiveness and sustainability of exogenous CGRP supplementation.

As its name “phosphatase of activated cells 1” suggests (32), PAC1 expression is significantly upregulated following immune cell activation. This characteristic has been validated in various types of immune cells (26, 27, 29). Here, our scRNA-Seq data also showed that *Pac1* expression tended to be enriched in activated ILC2s (Supplemental Figure 5C), possibly as a means to ensure they accept the proper regulation of CGRP. Interestingly, the upstream mechanisms regulating PAC1 expression show diversity. Studies by our group have found that p53 can directly bind to the promoter region of *PAC1*, promoting its expression (28); in CD4^+^ T cells, the *PAC1* gene can be silenced through methylation of the CpG island (27); in CD8^+^ T cells, EGR1, which is induced by ROS, plays a major role in promoting PAC1 expression (29). In this study, according to our scRNA-Seq analyses, the expression distribution of *Pac1* and *Egr1* in murine ILC2s was similar (Supplemental Figure 5C), suggesting that the EGR1/PAC1 axis may also function in ILC2s. Nevertheless, more evidence is still needed in the future. Additionally, it is worth noting that, although the mechanisms may differ, upregulated PAC1 plays a consistent inhibitory role in various immune cells, and thus it largely acts as a loyal immune checkpoint. It is therefore possible that PAC1 may emerge as a broad immunoregulatory drug target. Furthermore, unraveling the mechanisms behind the “checkpoint” property of PAC1 will provide a deeper understanding of the development and regulation of the immune system.

As a member of the nuclear-localized DUSP family, PAC1 was initially considered a classical MAPK phosphatase (26). However, as research advances, the diversity of the molecular functions of PAC1 are far beyond that originally imagined, and can even break away dependency on its classical phosphatase domain**.** In CD8^+^ T cells, we have shown that PAC1 can act as an epigenetic regulator, which reshapes chromatin accessibility by recruiting the Mi-2β nucleosome-remodeling deacetylase (NuRD) complex through its N-terminal domain, thus inhibiting the expression of antitumor effector genes (e.g., Gzmb) (29). Notably, research in recent years is also gradually revealing the important role of epigenetic modification in the functional regulation of ILC2s (33, 34). In the present study, we found that PAC1 was needed to increase the chromatin accessibility of CGRP-response genes in ILC2s, which proposed a new epigenetic regulator in ILC2s. Moreover, it seems that the mechanisms by which PAC1 selects specific chromatin regions to influence in different types of immune cells is also an interesting subject worth exploring in the future. On the other hand, we have also reported that when activated, induced PAC1 dephosphorylates Tyr^705^ of the transcription factor STAT3 in CD4^+^ T cells, thus suppressing their differentiation into Th17 cells (27). However, it is interesting to note that another group has reported that mice with sustained phosphorylation of STAT3 (Tyr^705^) in ILC2s exhibit lower papain-induced type 2 inflammation (35), theoretically contrasting with our findings, but supporting the idea that PAC1 does not act as a phosphatase of STAT3 in ILC2s. Our study again indicates that PAC1 functionality exhibits cellular heterogeneity. Exploring the distinctive roles of PAC1 in other types of immune cells may provide novel insights into the coordination of different immune cells during different immune responses.

## Methods

### Sex as a biological variable.

Our study examined male and female mice, and similar findings are reported for both sexes. Six- to 8-week-old sex- and age-matched mice were used for all animal experiments. For the human study, peripheral blood samples were obtained from both male and female participants.

### Mice.

*Pac1^–/–^* mice (C57BL/6J background) were generated as previously described (27). *Rag1^–/–^* mice (C57BL/6J background) and *Pac1^fl/fl^* mice (C57BL/6J background) were purchased from Jiangsu GemPharmatech Biotechnology Co. Ltd. *Rag2^–/–^* *Il2rg^–/–^* mice (C57BL/6J background) and B6(C)-*Il5*^tm1.1(icre)Lky^/J mice (Red5 mice) were a gift from Chao Zhong (Peking University, Peking, China). B6(C)-*Il5*^tm1.1(icre)Lky^/J mice (Red5 mice) can also be purchased from The Jackson Laboratory (stock no. 030926). All mice were kept in a specific pathogen–free (SPF) facility at Peking University Care Industrial Park with a 12-hour light/12-hour dark cycle, an ambient temperature of 20°C–24°C, and humidity of 30%–70%.

### Human samples.

Fresh human peripheral blood samples were obtained from the Department of Respiratory and Critical Care Medicine, Peking University Third Hospital.

### Preparation of cell suspensions from murine tissues.

Murine bone marrow cells were obtained by flushing bilateral femurs with 10 mL prechilled PBS. RBCs were lysed with ammonium chloride potassium (ACK) buffer.

Cells in murine BALF was collected by gently washing twice with 1 mL prechilled PBS through the main bronchus.

The isolation of cells from murine lungs was carried out by first euthanizing mice and immediately perfusing them with 20 mL PBS through the right ventricle to remove blood. Subsequently, lung lobes were removed, cut into small pieces, and digested in RPMI-1640 medium with 3 unit/mL dispase II (Roche) and 0.5 mg/mL DNase I (MilliporeSigma) for 1 hour at 37°C with continuous shaking. The crude suspensions were filtered through 100 μm cell strainers, and the remaining RBCs were lysed with ACK buffer.

For the isolation of cells from murine intestinal lamina propria and colonic lamina propria, small intestine and colon were collected from euthanized mice, and the contents were emptied. The tissues were then cut into pieces 1–2 cm in length and incubated for 20 minutes at 37°C in PBS containing 0.3% BSA, 5 mM EDTA, and 1 mM DTT. Next, the tissues were vortexed 3 times with PBS containing 2 mM EDTA to remove epithelial cells. The remaining tissues were digested for 45 minutes at 37°C in RPMI 1640 containing 0.05 mg/mL collagenase IV (Roche), 0.5 mg/mL dispase II, and 0.1 mg/mL DNase I. The crude suspensions were passed through a 100 μm cell strainer.

All tissue cells were washed and resuspended in FACS buffer (RPMI-1640 medium with 1% FBS) and filtered through 40 μm cell strainers before being used for subsequent experiments.

### Allergic airway inflammation model.

For *A. alternata*–induced allergic airway inflammation, each mouse was anesthetized, and 5 μg *A. alternata* (Geerlabs), combined with 1 μg CGRP (Anaspec) if needed, was administered intranasally once a day for 4 consecutive days. For papain-induced allergic airway inflammation, each mouse was anesthetized, and 25 μg papain (Merck) was administered intranasally once a day for 3 consecutive days. One day after the last administration, treated mice were euthanized and analyzed.

### In vivo ILC2 expansion and activation in lung.

Each mouse was anesthetized, and 500 ng recombinant IL-33 (BioLegend) was administered intranasally once a day for 4 consecutive days. One day after the last administration, the treated mice were euthanized and analyzed.

### In vivo expansion of inflammatory ILC2s in lung.

Each mouse was anesthetized, and 500 ng recombinant IL-25 (Sino Biological) was administered intranasally once a day for 4 consecutive days. One day after the last administration, the treated mice were euthanized and analyzed.

### Flow cytometry.

For surface marker staining, cells were preincubated with purified anti-CD16/anti-CD32 antibodies (Thermo Fisher Scientific, clone: 93) to block Fc receptors and subsequently incubated with specific antibodies for 45 minutes at room temperature.

For analysis of nuclear transcription factors, cells were first stained with antibodies against surface antigens and then fixed and permeabilized using a FOXP3/Transcription Factor Staining Buffer Set (Thermo Fisher Scientific), before incubation with antibodies recognizing the transcription factors indicated.

For measurement of intracellular cytokines, cells were stimulated in complete RPMI-1640 medium (10% FBS) containing 100 ng/mL PMA (MilliporeSigma), 500 ng/mL ionomycin (MilliporeSigma), and 1× brefeldin A (stock: 1,000×, Thermo Fisher Scientific) for 5 hours. Cells were subsequently surface stained, fixed, and permeabilized using an intracellular fixation and permeability kit (Thermo Fisher Scientific), and the cells were then incubated with antibodies recognizing the indicated cytokines.

All flow cytometric analyses were performed on a LSRFortessa (BD Biosciences), and the results were analyzed with FlowJo software (version 10).

Antibodies specific to mouse CD3ε (145-2C11), CD4 (GK1.5), CD5 (53-7.3), CD8α (53-6.7), CD11b (M1/70), CD11c (N418), CD19 (eBio1D3), CD45 (30-F11), F4/80 (BM8), CD90.2 (53-2.1), NK1.1 (PK136), ST2 (RMST2-2), EpCAM (G8.8), SCA-1 (D7), FLT3 (A2F10), Ly6G (1A8), integrin α4β7 (DATK32), FcεR1α (MAR-1), MHC-II (M5/114.15.2), Ki67 (SolA15), KLRG1 (2F1), and IL-13 (eBio13A) were purchased from Thermo Fisher Scientific. Antibodies specific to the human hematopoietic lineage markers CD127 (eBioRDR5) and CRTH2 (BM16) were purchased from Thermo Fisher Scientific. Antibodies specific to mouse CD45.1 (A20), CD45.2 (104), CD127 (A7R34), IL-25R (9B10), CD25 (3C7), Ly6C (HK1.4), c-KIT (ACK2), GATA3 (16E10A23), T-bet (4B10), and IL-5 (TRFK5) were purchased from BioLegend. Antibodies specific to mouse Siglec-F (E50-2440) and RORγT (Q21-559) were purchased from BD Biosciences. Fixable cell viability dye was purchased from Thermo Fisher Scientific.

### Adoptive transfer of ILC2s.

Lung ILC2s sorted from CD45.2^+^ *Pac1^+/+^* mice and CD45.1^+^ *Pac1^–/–^* mice were mixed at a 1:1 ratio and then injected intravenously into *Rag2^–/–^* *Il2rg^–/–^* mice (1×10^4^ ILC2s/mouse). Twenty-four hours later, the recipient mice were challenged with IL-33 (intranasally) once a day for 4 consecutive days. Mice were euthanized and analyzed 24 hours after the last challenge.

### Bone marrow chimera.

Bone marrow cells from CD45.2^+^ *Pac1^+/+^* and CD45.1^+^ *Pac1^–/–^* mice were mixed at a 1:1 ratio and then injected intravenously into *Rag2^–/–^* *Il2rg^–/–^* mice (4 × 10^6^ cells/mouse) that had been subjected to myeloablation by administration of busulfan (MilliporeSigma). The total dose of busulfan was 90 mg/kg body weight, which was achieved by 3 intraperitoneal injections. After an 8-week reconstitution, the recipient mice were challenged with IL-33 (intranasally) once a day for 4 consecutive days. Mice were euthanized and analyzed 24 hours after the last challenge.

### Cell sorting.

All cell sorting was performed using a BD FACSAria III cell sorter (BD Bioscience), with purity ensured to be greater than 90%. Before murine ILC2 sorting, lung cells were predepleted of most T cells, B cells, myeloid cells, NK cells, and erythroid cells through immunomagnetic selection using an EasySep Mouse ILC2 Enrichment Kit (STEMCELL Technologies).

### In vitro ILC2 cell culture.

Sorted murine lung ILC2s were incubated in complete RPMI-1640 medium (50 μM β-mercaptoethanol, 10% FBS and 1% penicillin/streptomycin), supplemented with 50 ng/mL IL-2 (BioLegend) and 100 ng/mL IL-7 (BioLegend). After expansion for 1–2 weeks, IL-2 and IL-7 were removed and ILC2s were cultured in 96-well plates at a density of 5,000–10,000 cells per well for further functional analyses. Various stimuli were added at the following concentrations: IL-7 (100 ng/mL, BioLegend), IL-33 (100 ng/mL, BioLegend), CGRP (500 ng/mL, Anaspec), and NMU (500 ng/mL, MCE). After the indicated stimulation duration, cell culture supernatants were collected for cytokine analyses, and ILC2s were collected for subsequent experiments.

### Quantitative real-time PCR.

Total RNA from sorted immune cells or lung tissues was extracted using a RaPure Total RNA Micro kit (Magen) and reverse transcribed using 5× All-in-One RT Master Mix (ABM). For mouse or human ILC2s, live cells were sorted directly into the lysis buffer of a Single Cell Full Length mRNA Amplification Kit (Vazyme). mRNAs were then reverse transcribed to cDNAs according to the manufacturer’s protocols. The reverse transcription products were amplified with Taq Pro Universal SYBR qPCR Master Mix (Vazyme) and analyzed with gene-specific primers on an ABI 7500 system (Thermo Fisher Scientific). The sequences of the qPCR primers used in this study are listed in Supplemental Table 1.

### Cytokine analyses.

For cytokine analyses of the purified ILC2 culture supernatants, multianalyte profiling was performed using a ProcartaPlex Multiplex Immunoassay System (Thermo Fisher Scientific) according to the manufacturer’s instructions. The panel simultaneously measured protein levels of murine IL-1β, IL-2, IL-4, IL-5, IL-6, IL-9, IL-10, IL-12p70, IL-13, IL-17A, IL-18, IL-22, IL-23, IL-27, GM-CSF, TNF-α, and IFN-γ.

### scRNA-Seq.

Lung ILC2s sorted from *Pac1^+/+^* or *Pac1^–/–^* mice (~13,000 ILC2s from mixed lung cell samples from 4–5 mice, cell viability >98%) were encapsulated into droplets, and libraries were prepared using a Chromium Next GEM Single Cell 3′ GEM, Library and Gel Bead Kit, version 3.1 (10X Genomics), according to the manufacturer’s protocol. The scRNA-Seq libraries generated were sequenced on an Illumina Novaseq 6000 platform (2 × 150 bp). After sequencing, the reads were processed using Cell Ranger (10X Genomics, version 6.1.1) for demultiplexing, alignment to the *Mus musculus* mm10 genome, barcode processing, and cell quality control, ultimately generating a feature-barcode matrix. The feature-barcode matrices of WT and *Pac1^–/–^* ILC2s were further analyzed in R (version 4.2.3), and the main R packages included Seurat (version 3), AUCell (for cell scoring using gene sets), and Monocle (version 3, for the pseudotime analysis).

### Bulk mRNA-Seq.

Lung ILC2s from *Pac1^+/+^* or *Pac1^–/–^* mice were treated under different conditions of stimulation for 4 hours, and then approximately 500 ILC2s were sorted directly into the lysis buffer of a Single Cell Full Length mRNA-Amplification Kit (Vazyme). mRNAs in the samples were then reverse transcribed to cDNAs and amplified according to the manufacturer’s protocols. The sequencing libraries were established using a TruePrep DNA Library Prep Kit V2 for Illumina (Vazyme), according to the manufacturer’s instructions. The quality of the sequencing libraries was assessed using the Agilent 2100 Bioanalyzer system, and the sequencing was performed on a NovaSeq platform with paired-end 150 bp reads (Illumina). The RNA-Seq reads were aligned to the *Mus musculus* mm10 genome using HISAT2, and differential expression analyses were performed using R package DESeq2 (version 1.24.0). GSEA was performed using the software from the Broad Institute (version 4.3.2), and Gene Ontology (GO) databases were used.

### Bulk fast ATAC-Seq.

Lung ILC2s from *Pac1^+/+^* or *Pac1^–/–^* mice (~50,000 ILC2s) were treated under different conditions of stimulation for 6 hours, and the ATAC-Seq libraries were established using a Hyperactive ATAC-Seq Library Prep Kit for Illumina (Vazyme), according to the manufacturer’s instructions. The sequencing was then performed on a NovaSeq platform with paired-end 150 bp reads (Illumina). The reads were aligned to the *Mus musculus* mm10 genome, and the peaks were called using MACS2 (version 2.1.2). Visualization of peak distribution along genomic regions of the genes of interest was performed on the Integrative Genomics Viewer (IGV, version 2.16.2).

### In vitro cell proliferation assay.

Lung ILC2s from *Pac1^+/+^* or *Pac1^–/–^* mice were labeled with CellTrace Violet (Thermo Fisher Scientific) according to the manufacturer’s instructions and subsequently cultured under different conditions of stimulation for 3 days. Expression of CellTrace Violet in live ILC2s was analyzed by flow cytometry.

### Measurement of cAMP production.

Lung ILC2s from *Pac1^+/+^* or *Pac1^–/–^* mice were cultured in 96-well plates at a density of 5,000–10,000 cells per well and subsequently treated under different conditions of stimulation for 20 minutes. The intracellular cAMP concentrations were measured using a Screen Quest Fluorimetric ELISA cAMP Assay Kit (AAT Bioquest), according to the manufacturer’s instructions.

### Histological analyses.

Murine lung tissues were fixed for at least 48 hours with 10% neutral formalin (Leagene), followed by paraffin embedment and slicing. H&E staining was performed using a Leica Autostainer XL. PAS staining was performed using a Glycogen Periodic Acid Schiff Stain Kit (Solarbio). An Olympus Microscope IX53 was used to obtain the histological staining images. To quantify the severity of airway inflammation, we invited a pathologist from Peking University Health Science Center to score all the slides (including H&E and PAS staining) in a blinded manner. The evaluation metrics included the extent of inflammation around the bronchi and blood vessels, the hyperplasia state of goblet cells, and the amount of interstitial infiltrate (21, 24). The scoring criteria were as follows: 0, none; 1, mild; 2, moderate; 3, marked; 4, severe; 5, profound, severe.

### Statistics.

Statistical analyses were performed using GraphPad Prism (version 9.5.1, GraphPad Software). All data are presented as the mean ± SEM. To assess differences between 2 groups, a 2-tailed, unpaired or paired Student’s *t* test was used. To compare intergroup differences, a 2-way ANOVA followed by a Holm-Šidák multiple-comparison test was used. A *P* value of less than 0.05 was considered statistically significant.

### Study approval.

All mouse experimental procedures were approved and monitored by the Animal Care and Use Committee of Peking University (LA2020273). All procedures related to human blood samples were conducted under the approval of the Ethics Committee of Peking University Third Hospital (IRB00006761‑M2021339), and informed patient consent was obtained from all individuals in advance of sample collection.

### Data availability.

The data and materials that support the findings of this study are available from the corresponding author upon reasonable request. All next-generation sequencing data were deposited in the NCBI’s Gene Expression Omnibus (GEO) database (GSE272724 for scRNA-Seq data, GSE272723 for bulk mRNA-Seq data, and GSE272722 for fast ATAC-Seq data). A Supporting Data Values file has been provided for all numerical data. This study did not generate any unique codes.

## Author contributions

Yuan Jin designed and performed the experiments, analyzed data, and wrote and revised the manuscript. BL performed the experiments and participated in the revision of the manuscript. QL provided clinical materials. XM and XT helped with the scRNA-Seq experiment. Yan Jin supervised the experiments. YY conceived this research, supervised the experiments, and wrote the manuscript.

## Supplementary Material

Supplemental data

Supporting data values

## Version 1 09/17/2024

In-Press Preview

## Version 2 11/01/2024

Electronic publication
