# Supplementary material for: PAC1 constrains type 2 inflammation through promotion of CGRP signaling in ILC2s
Source: J Clin Invest. 2024 Sep 17;134(21):e180109. doi: 10.1172/JCI180109 (PMC11527444; doi:10.1172/JCI180109)
Supplement: Supplemental data [file jci-134-180109-s214.pdf]

Supplemental Figure 1

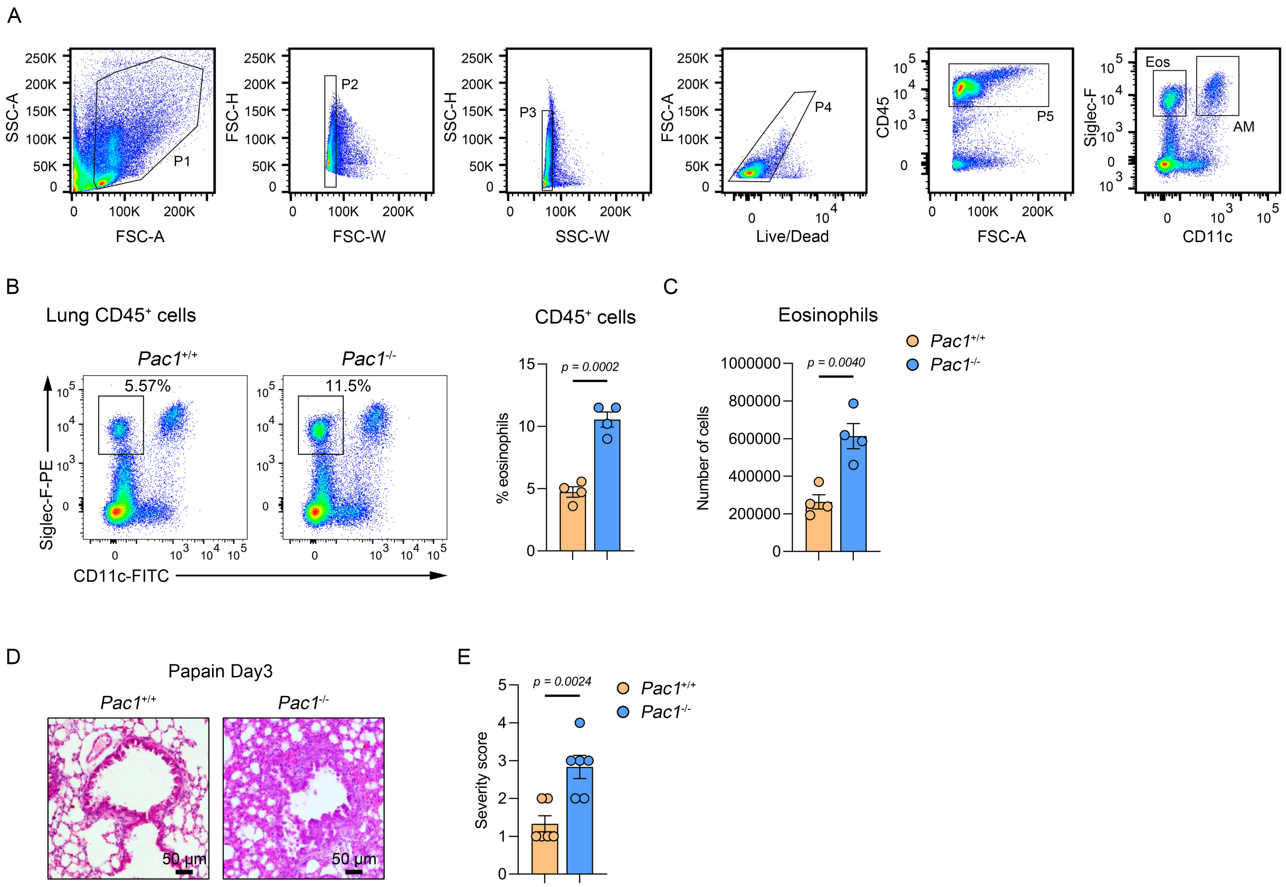

Supplemental Figure 1. PAC1 constrains papain-induced allergic airway inflammation in mice

(A) Flow cytometry plots showing the gating strategies to identify eosinophils in murine BALF or lungs.

(B-C) Frequency **(B)** and absolute number **(C)** of lung eosinophils in *Pac1*$^{+/+}$ (n = 4) and *Pac1*$^{-/-}$ mice (n = 4) on day 3 after papain administration, as determined by flow cytometry.

(D) Representative H&E-stained lung sections from *Pac1*$^{+/+}$ and *Pac1*$^{-/-}$ mice on day 3 after papain administration.

(E) The histological score of lungs from *Pac1*$^{+/+}$ (n = 6) and *Pac1*$^{-/-}$ mice (n = 6) on day 3 after papain administration.

Data are shown as the means ± SEM. Statistical significance was assessed using a two-tailed unpaired Student's t test.

Supplemental Figure 2

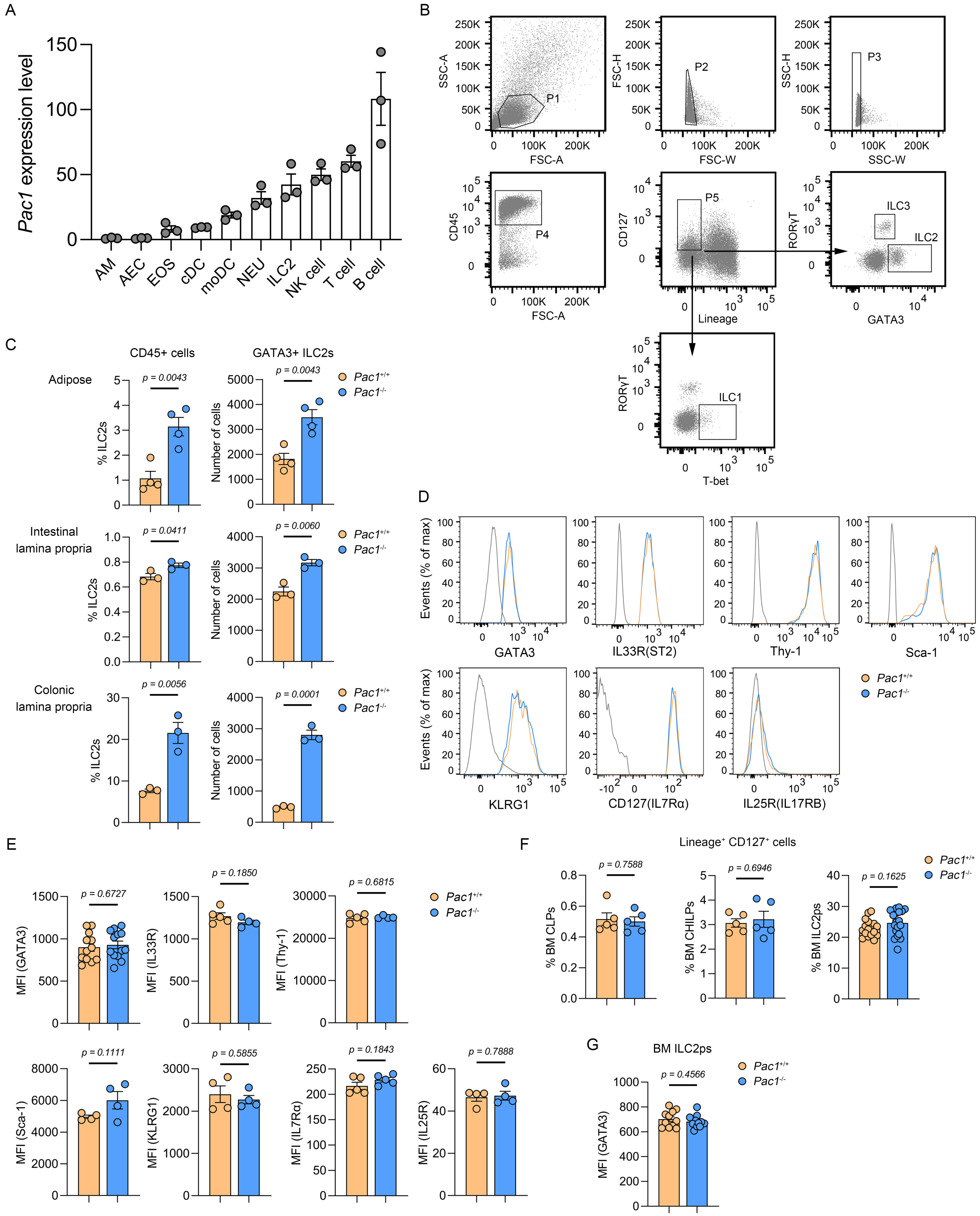

Supplemental Figure 2. PAC1 is not involved in affecting the phenotype and heterogeneity of ILC2s, and does not affect the developmental processes of ILC2s

(A) *Pac1* expression levels in various types of cells sorted from murine lungs, as determined by qPCR. Data are shown as the means ± SEM. AM, alveolar macrophages; AEC, alveolar epithelial cells; EOS, eosinophils; cDC, conventional dendritic cells; moDC, monocyte-derived dendritic cells; NEU, neutrophils.

(B) Flow cytometry plots showing the gating strategies to identify T-bet$^+$ ILC1s, GATA3$^+$ ILC2s and RORγT$^+$ ILC3s in murine lungs.

(C) Frequency and absolute number of GATA3$^+$ ILC2s in epididymal adipose tissue, intestinal lamina propria and colonic lamina propria of *Pac1*$^{+/+}$ (n = 3-4) and *Pac1*$^{-/-}$ mice (n = 3-4) in their resting states, as determined by flow cytometry.

(D-E) Expression histograms **(D)** of selected ILC2 markers in *Pac1*$^{+/+}$ and *Pac1*$^{-/-}$ lung GATA3$^+$ ILC2s in a resting states and mean fluorescence intensity (MFI) of each marker analyzed in **(E)**, as determined by flow cytometry.

(F) Frequency of CLPs (*Pac1*$^{+/+}$, n = 5; *Pac1*$^{-/-}$, n = 5), CHILPs (*Pac1*$^{+/+}$, n = 5; *Pac1*$^{-/-}$, n = 5), and ILC2ps (*Pac1*$^{+/+}$, n = 17; *Pac1*$^{-/-}$, n = 19) in bone marrow of *Pac1*$^{+/+}$ and *Pac1*$^{-/-}$ mice in their resting state, as determined by flow cytometry.

(G) MFI of GATA3 in bone marrow ILC2ps (*Pac1*$^{+/+}$, n = 11; *Pac1*$^{-/-}$, n = 11) of *Pac1*$^{+/+}$ and *Pac1*$^{-/-}$ mice in their resting state, as determined by flow cytometry.

Data are shown as the means ± SEM. Statistical significance was assessed using a two-tailed unpaired Student’s t test.

Supplemental Figure 3

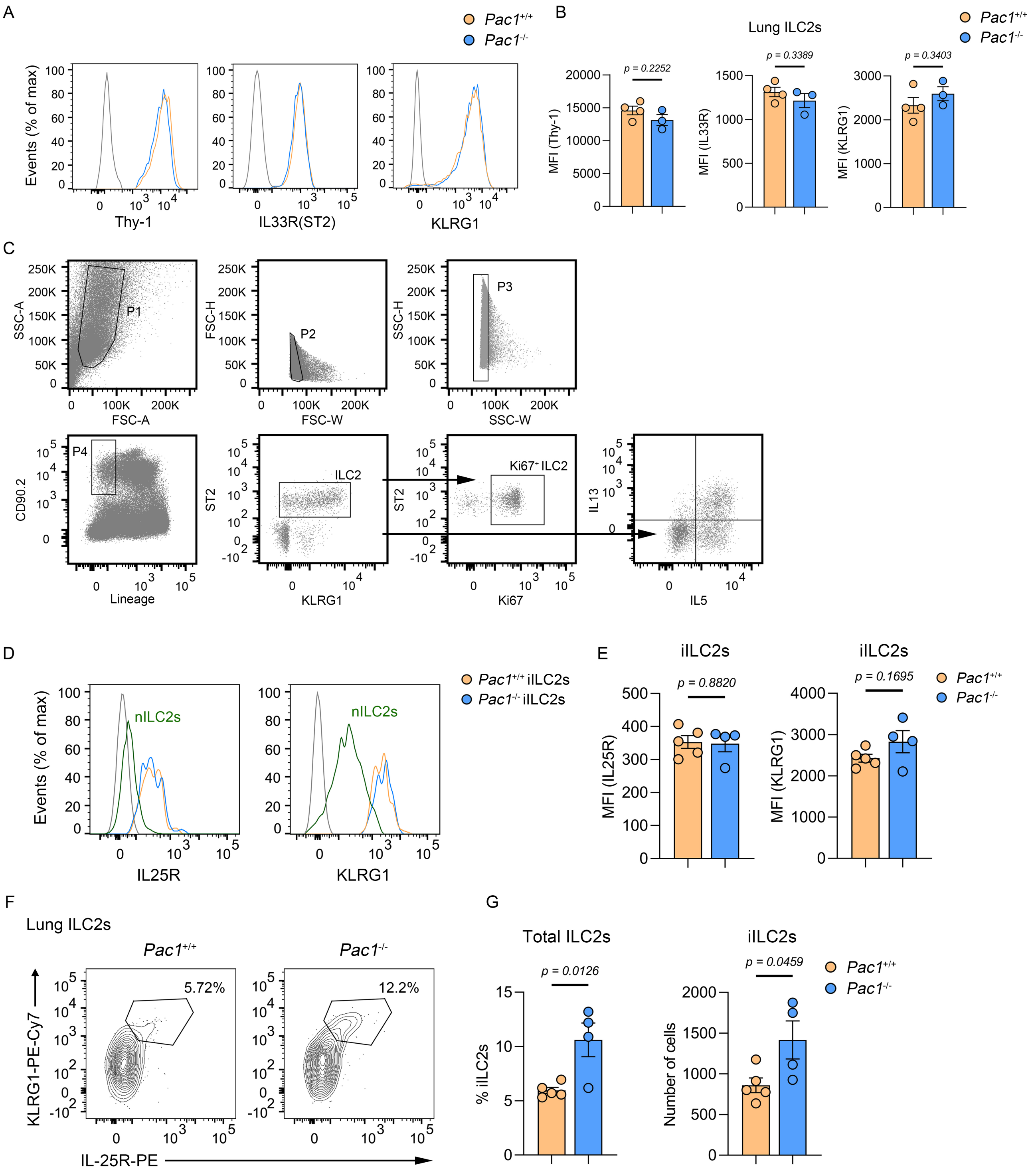

Supplemental Figure 3. PAC1 also exhibits suppressive effects on inflammatory ILC2s

(A-B) Expression histograms **(A)** of selected ILC2 markers in *Pac1*$^{+/+}$ ($n = 4$) and *Pac1*$^{-/-}$ ($n = 3$) lung GATA3^{+} ILC2s on day 4 after IL-33 administration and mean fluorescence intensity (MFI) of each marker analyzed in **(B)**, as determined by flow cytometry.

(C) Flow cytometry plots showing the gating strategies to identify ILC2s, Ki67^{+} ILC2s and IL-5^{+} IL-13^{+} ILC2s in murine lungs.

(D-E) Expression histograms **(D)** of IL-25R and KLRG1 in *Pac1*$^{+/+}$ ($n = 5$) and *Pac1*$^{-/-}$ ($n = 4$) lung inflammatory ILC2s (iILC2s) on day 4 after IL-25 administration and mean fluorescence intensity (MFI) of each marker analyzed in **(E)**, as determined by flow cytometry.

(F-G) Frequency **(F)** and absolute number **(G)** of lung iILC2s in *Pac1*$^{+/+}$ ($n = 5$) and *Pac1*$^{-/-}$ mice ($n = 4$) on day 4 after IL-25 administration, as determined by flow cytometry.

Data are shown as the means ± SEM. Statistical significance was assessed using a two-tailed unpaired Student's t test.

Supplemental Figure 4

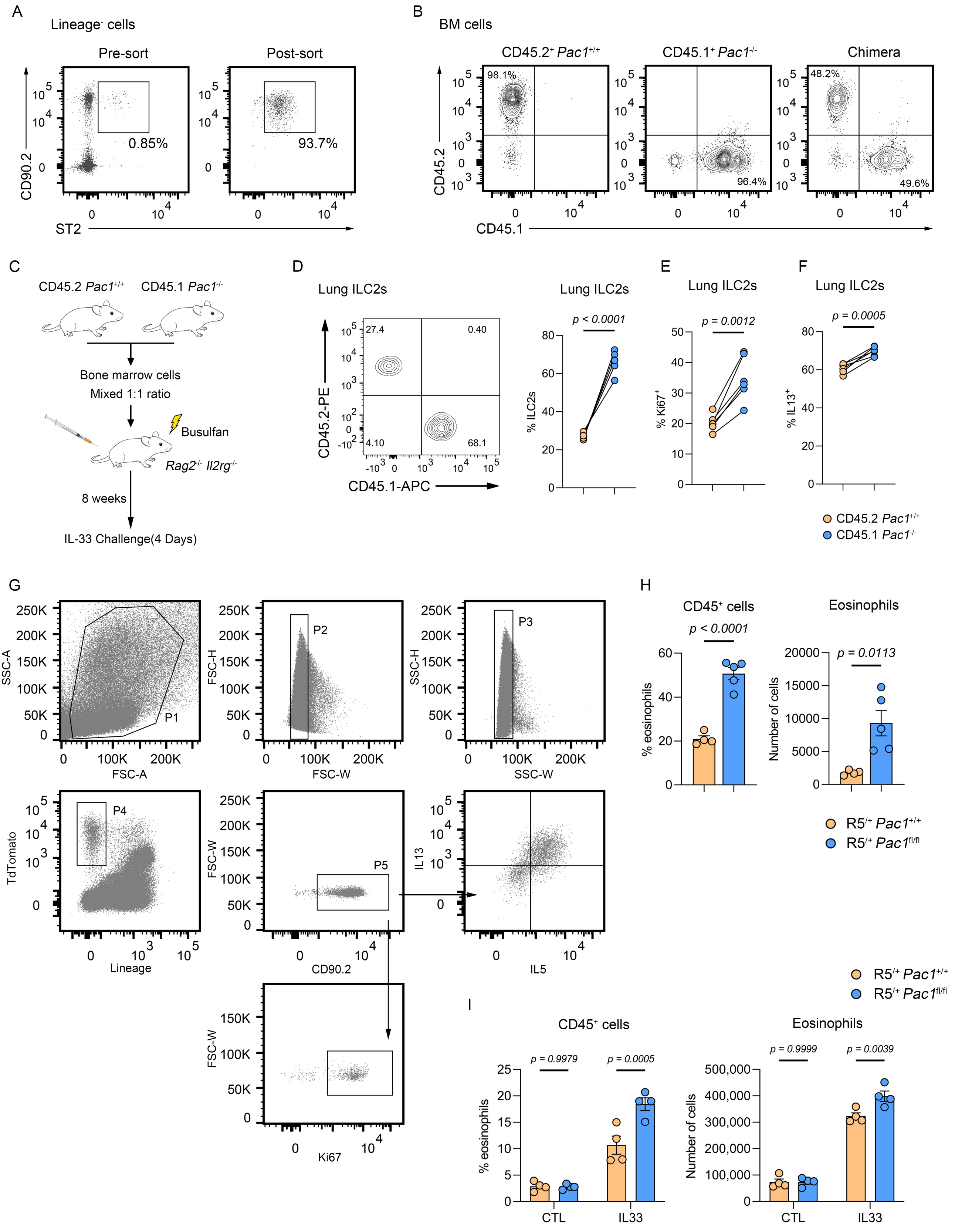

Supplemental Figure 4. PAC1 plays a cell-intrinsic inhibitory role in ILC2s

(A) Pre-sort and post-sort purity of ILC2s from murine lungs.

(B) Frequency of CD45.1$^+$ cells and CD45.2$^+$ cells in donor CD45.2$^+$ *Pac1*$^{+/+}$ mice, donor CD45.1$^+$ *Pac1*$^{-/-}$ mice and recipient *Rag2*$^{-/-}$ *Il2rg*$^{-/-}$ mice in the bone marrow chimera model.

(C) Experimental protocol followed for generating mixed bone marrow chimera.

(D) Frequency of lung CD45.2$^+$ *Pac1*$^{+/+}$ ILC2s and CD45.1$^+$ *Pac1*$^{-/-}$ ILC2s in chimeric mice (n = 6) on day 4 after IL-33 administration, as determined by flow cytometry.

(E) Frequency of lung CD45.2$^+$ *Pac1*$^{+/+}$ Ki67$^+$ ILC2s and CD45.1$^+$ *Pac1*$^{-/-}$ Ki67$^+$ ILC2s in chimeric mice (n = 6) on day 4 after IL-33 administration, as determined by flow cytometry.

(F) Frequency of lung CD45.2$^+$ *Pac1*$^{+/+}$ IL-13$^+$ ILC2s and CD45.1$^+$ *Pac1*$^{-/-}$ IL-13$^+$ ILC2s in chimeric mice (n = 6) on day 4 after IL-33 administration, as determined by flow cytometry.

(G) Flow cytometry plots showing the gating strategies to identify Ki67$^+$ ILC2s and IL-5$^+$ IL-13$^+$ ILC2s in the lungs of R5$^{/+}$ mice.

(H) Frequency and absolute number of BALF eosinophils in R5$^{/+}$ *Pac1*$^{+/+}$ (n = 4) and R5$^{/+}$ *Pac1*$^{fl/fl}$ mice (n = 5) on day 4 after IL-33 administration, as determined by flow cytometry.

(I) Frequency and absolute number of lung eosinophils in R5$^{/+}$ *Pac1*$^{+/+}$ and R5$^{/+}$ *Pac1*$^{fl/fl}$ mice in resting state (R5$^{/+}$ *Pac1*$^{+/+}$, n = 4; R5$^{/+}$ *Pac1*$^{fl/fl}$, n = 4) or on day 4 after IL-33 administration (R5$^{/+}$ *Pac1*$^{+/+}$, n = 4; R5$^{/+}$ *Pac1*$^{fl/fl}$, n = 4), as determined by flow cytometry.

Data are shown as the means ± SEM. Statistical significance was assessed using a two-tailed paired Student's t test (**D-F**), a two-way ANOVA followed by Holm-Sidak multiple-comparisons test (**I**) or a two-tailed unpaired Student's t test (**H**).

Supplemental Figure 5

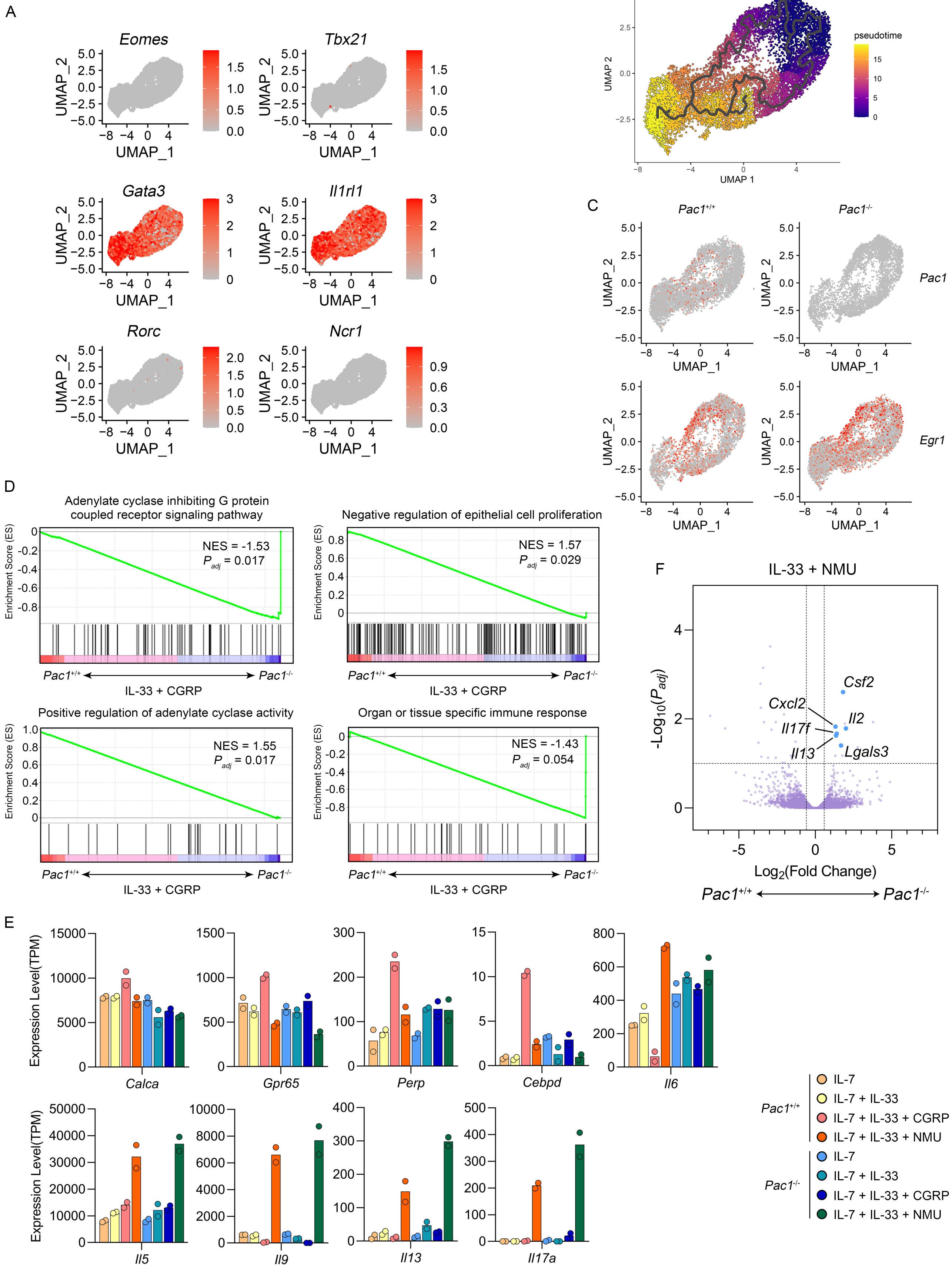

Supplemental Figure 5. PAC1 deficiency impairs CGRP signaling in ILC2s

(A) Expression distribution of classic marker genes of ILC2s (*Gata3*, *Il1rl1*) or other ILC subtypes (*Eomes*, *Tbx21*, *Rorc*, *Ncr1*) in sorted murine lung ILC2s.

(B) The regulation trajectory of murine lung ILC2s displayed according to a pseudotime analysis, using the R package Monocle 3.

(C) Expression distribution of *Pac1* and *Egr1* in $Pac1^{+/+}$ and $Pac1^{-/-}$ lung ILC2s.

(D) GSEA plots showing representative pathways enriched in $Pac1^{+/+}$ and $Pac1^{-/-}$ murine lung ILC2s after treatment with IL-33 plus CGRP. NES, normalized enrichment score.

(E) Expression levels (transcripts per million, TPM) of representative genes in $Pac1^{+/+}$ and $Pac1^{-/-}$ murine lung ILC2s under four different conditions of stimulation. Two replicates were analyzed per condition.

(F) Volcano plots of DEGs in $Pac1^{+/+}$ versus $Pac1^{-/-}$ murine lung ILC2s after treatment with IL-33 plus NMU (Fold Change > 1.5; $P_{adj} < 0.05$). Representative DEGs are shown.

Supplemental Figure 6

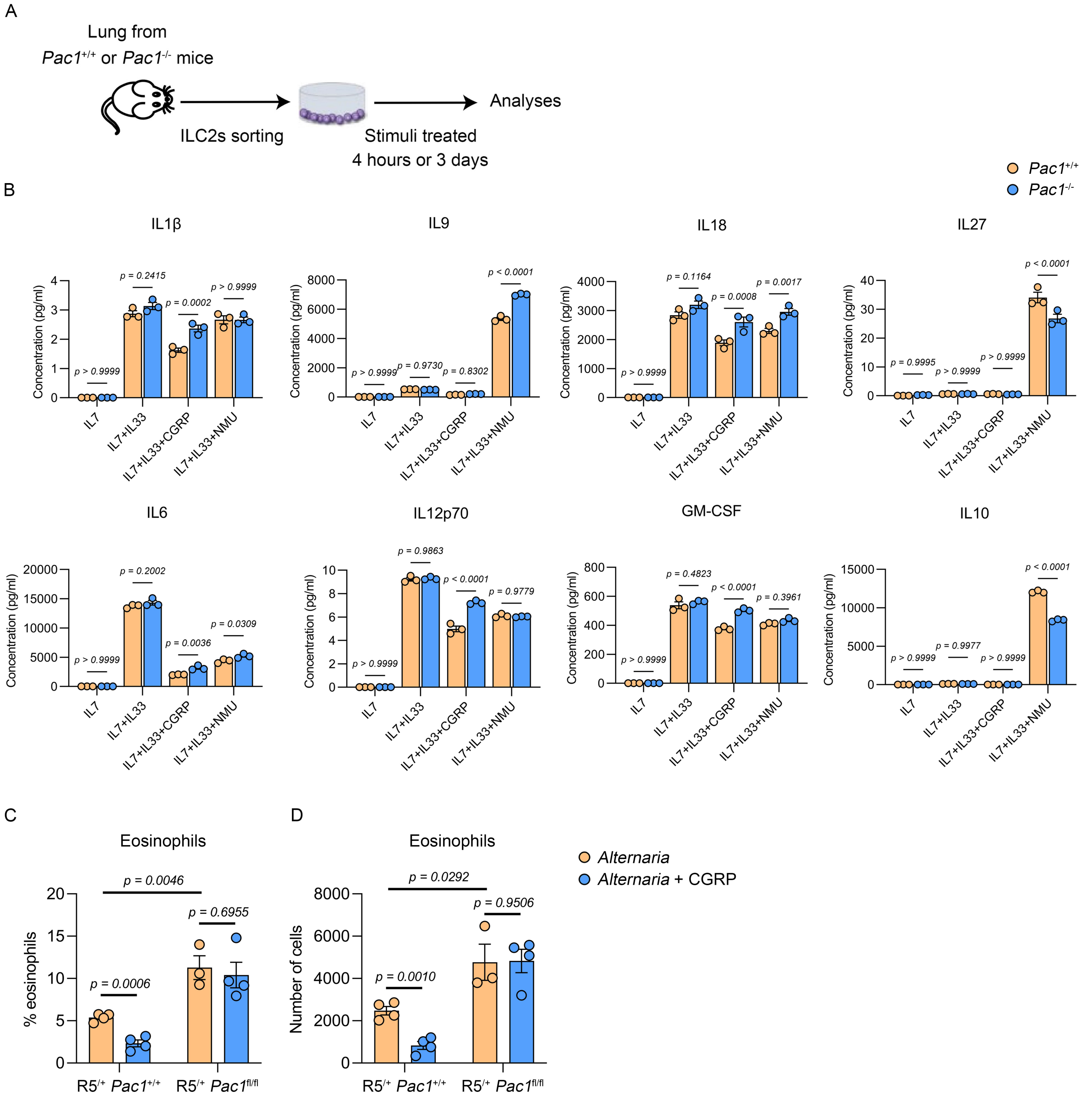

Supplemental Figure 6. PAC1 promotes CGRP-mediated inhibition of ILC2 responses

(A) Strategy for *in vitro* ILC2 stimulation.

(B) Purified *Pac1*$^{+/+}$ and *Pac1*$^{-/-}$ murine lung ILC2s were treated under different conditions of stimulation for 3 days, and then the concentrations of a range of cytokines in the cell supernatants were assessed by Luminex liquid chip technology. Three replicates were analyzed per condition.

(C-D) Frequency **(C)** and absolute number **(D)** of BALF eosinophils in R5$^{/+}$ *Pac1*$^{+/+}$ and R5$^{/+}$ *Pac1*$^{fl/fl}$ mice on day 4 after *Alternaria alternata* administration (R5$^{/+}$ *Pac1*$^{+/+}$, $n = 4$; R5$^{/+}$ *Pac1*$^{fl/fl}$, $n = 4$) or *Alternaria alternata* plus CGRP administration (R5$^{/+}$ *Pac1*$^{+/+}$, $n = 4$; R5$^{/+}$ *Pac1*$^{fl/fl}$, $n = 4$), as determined by flow cytometry.

Data are shown as the means ± SEM. Statistical significance was assessed using a two-way ANOVA followed by Holm-Sidak multiple-comparisons test (**B**) or a two-tailed unpaired Student's t test (**C-D**).

Supplemental Table 1. The sequences of qPCR primers used in this study

	Forward primer (5'-3')	Reverse primer (5'-3')
mouse *Il5*	GACAAGCAATGAGACGATGAGG	CCACTCTGTACTCATCACACC
mouse *Il13*	TGGTATGGAGTGTGGACCTG	AGCAAAGTCTGATGTGAGAAAGG
mouse *Pac1*	TGCCGTGGTGCTGGATGAAA	CCTCGGGTCAGAGTTGCTATTT
mouse *Calca*	GCCTTTGAGGTCAATCTTGGA	TGGGAACAAAGTTGTCCTTCAC
mouse *Actb*	GAGACCTTCAACACCCCAGC	ATGTCACGCACGATTTCCC
human *CALCA*	CTCCATGCAGCACCATTCAG	GTGTGAAACTTGTTGAAGTCCTG
human *PAC1*	CAAGAGTATCCCTGTGGAGGAC	GAAACTGAAGTTGGGGGAGATG
human *IL13*	GAATCCCTGATCAACGTGTC	GAATCTGCAACTTCAATAGTCAGG
human *GPR65*	CGGAAGAAATATGGAAGGAAAGG	TTACACAGATATCAGCAGTTGG
human *GAPDH*	ACCCACTCCTCCACCTTTGA	ACCCACTCCTCCACCTTTGA
